# Intriguing Heteroleptic Zn^II^ bis(dipyrrinato) Emitters in the Far-Red Region With Large Pseudo-Stokes Shift for Bioimaging

**DOI:** 10.3389/fchem.2021.754420

**Published:** 2021-09-23

**Authors:** Roberta Tabone, Dominik Feser, Enrico D. Lemma, Ute Schepers, Claudia Bizzarri

**Affiliations:** ^1^ Institute of Organic Chemistry, Karlsruhe Institute of Technology (KIT), Karlsruhe, Germany; ^2^ Institute of Functional Interfaces (IFG), KIT, Eggenstein-Leopoldshafen, Germany; ^3^ Zoological Institute, Cell and Neurobiology, KIT, Karlsruhe, Germany

**Keywords:** bis(dipyrrinato) Zn ^II^ complexes, cell-viability, far-red emission, heteroleptic Zn ^II^ complexes, large Stokes shift, live-cell imaging, multiplexing

## Abstract

Novel heteroleptic Zn^II^ bis(dipyrrinato) complexes were prepared as intriguing emitters. With our tailor-made design, we achieved far-red emissive complexes with a photoluminescence quantum yield up to 45% in dimethylsulfoxide and 70% in toluene. This means that heteroleptic Zn^II^ bis(dipyrrinato) complexes retain very intense emission also in polar solvents, in contrast to their homoleptic counterparts, which we prepared for comparing the photophysical properties. It is evident from the absorption and excitation spectra that heteroleptic complexes present the characteristic features of both ligands: the plain dipyrrin (L_p_) and the π-extended dipyrrin (L_π_). On the contrary, the emission comes exclusively from the π-extended dipyrrin L_π_, suggesting an interligand nonradiative transition that causes a large *pseudo*-Stokes shift (up to 4,600 cm^−1^). The large *pseudo*-Stokes shifts and the emissive spectral region of these novel heteroleptic Zn^II^ bis(dipyrrinato) complexes are of great interest for bioimaging applications. Thus, their high biocompatibiliy with four different cell lines make them appealing as new fluorophores for cell imaging.

## Introduction

Far-red and near-infrared (NIR) fluorophores are highly desired probes for bioimaging and sensing applications in living organisms. In fact, they emit in the so-called “*biological imaging window*”, where interferences from absorbance by water and proteins and intrinsic autofluorescence are minimal (Weissleder, 2001; Hilderbrand and Weissleder, 2010). Nevertheless, a proper design of far-red/NIR dyes is necessary, as those probes usually suffer from photo-bleaching and low photoluminescence quantum yield (Φ) (Guo et al., 2014). Borondipyrromethene based dyes (BODIPYs) are among the most widely used fluorophore classes used in bioimaging. (Ni and Wu, 2014; Kowada et al., 2015; Grossi et al., 2016; Callaghan et al., 2019; Filatov, 2019; Kaur and Singh, 2019; Qu et al., 2019; Deng et al., 2021). The development of emissive bis(dipyrrinato) zinc complexes have received an increasing momentum only recently, in contrast to BODIPYs, as they were used mainly for supramolecular architectures and coordination polymers (Baudron, 2013; Matsuoka and Nabeshima, 2018; Jiang et al., 2020). With an appropriate design, bright fluorescence can also be achieved from Zn^II^ bis(dipyrrinato) complexes (I.V. Sazanovich, 2004). Even so, homoleptic zinc complexes suffer from an intramolecular electron transfer between the two electronically degenerate excited states of the identical dipyrrins. This process causes the population of a non-emissive symmetry-breaking charge transfer state (SBCT) (Trinh et al., 2014). Although SBCT is very appealing in potential applications such as artificial photosynthesis (Alqahtani et al., 2019; Tungulin et al., 2019) or photovoltaics, (Bartynski et al., 2015), it is not advantageous for other applications such as imaging, where high emission also in polar solvents is of utmost importance. A strategy to control this obstacle is encapsulation in nanoparticles (e.g. mesoporous silica) (Sani et al., 2020). Very recently, green-emitting homoleptic bis(dipyrrinato) zinc complexes were employed as selective probes for cancer cells and as photodynamic therapy photosensitisers (Karges et al., 2019a; Karges et al., 2019b; Karges et al., 2020). However, because of their homoleptic nature, encapsulation in polymeric nanoparticles was necessary to overcome the quenching effects in water.

In heteroleptic bis(dipyrrinato) zinc complexes, the electronically excited states of the two dipyrrinato ligands are energetically different. Thus, the absence of degeneracy sets aside the charge-separated state and these complexes are emissive in polar solvents. Our strategy focused on heteroleptic Zn^II^ bis(dipyrrinato) complexes that also benefit from a *pseudo*-Stokes shift (Kusaka et al., 2012; Sakamoto et al., 2016). Although Stokes shifts are defined as the separation in energy between the maxima in absorption and emission of a fluorophore, a *pseudo*-Stokes shift is associated with the difference between the emission and a relative maximum in absorption for an upper-lying excited state, which undergoes a radiation-less deactivation in favour to the lower (and emissive) excited state. Fluorophores with large (*pseudo*)-Stokes shifts are highly desirable in biochemical experiments so that the label emission is at a significant longer wavelength than excitation (e.g. intracellular imaging enabling multiplexing) (Rauf et al., 2010; Jeong et al., 2011; Shcherbakova et al., 2012; Holzapfel et al., 2018). Our new heteroleptic Zn^II^ bis(dipyrrinato) complexes herein presented have intriguing properties to be used as fluorescent emitters for bioimaging.

## Results and Discussion

**FIGURE 1:**
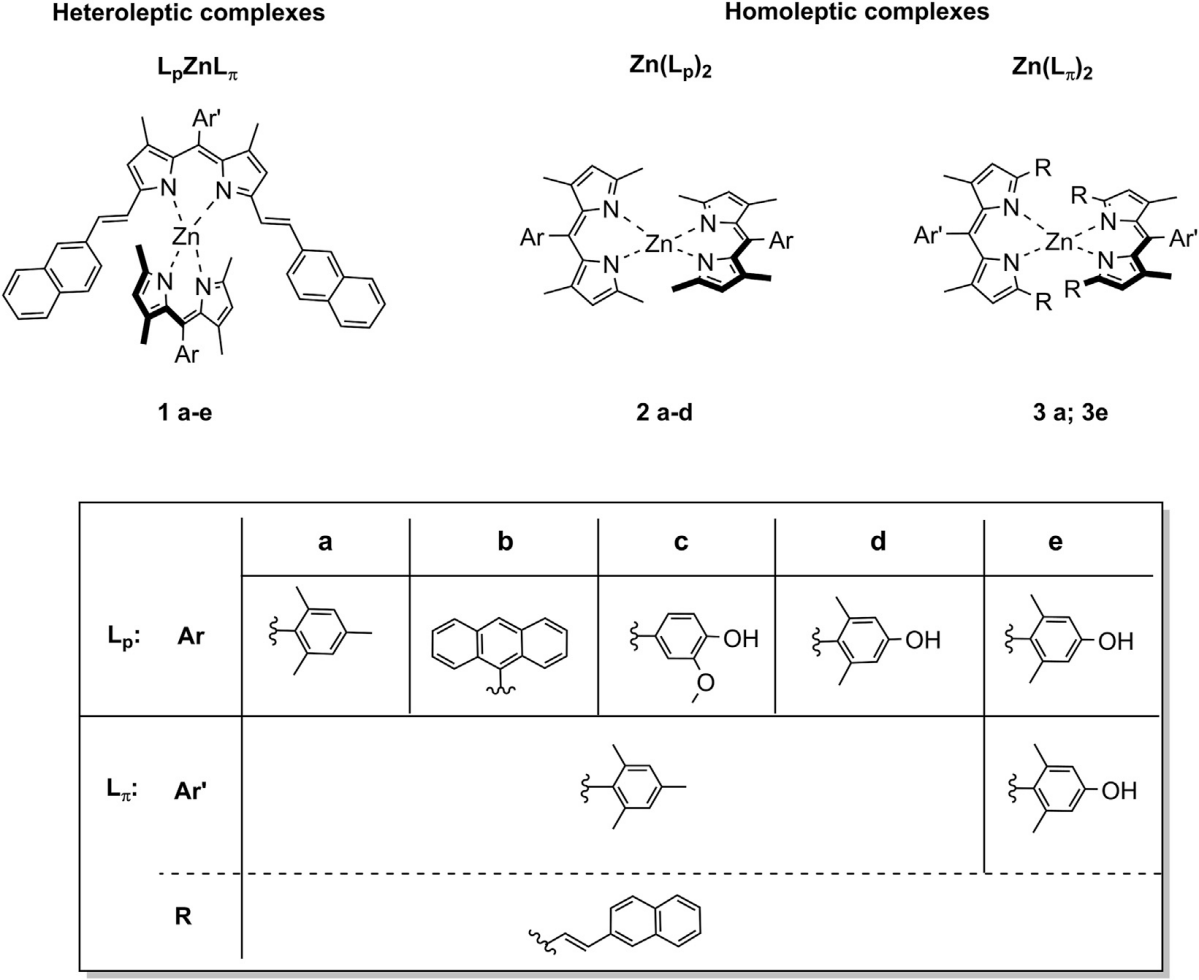
Chemical structures of the new heteroleptic Zn bis(dipyrrinato) complexes **1a-e** presented in this work. For comparison we reported also the study of the homoleptic complexes of type Zn (L_p_)_2_ **2a-d** and of type Zn (L_π_)_2_ **3a** and **3e**.

The synthesis of the plain dipyrrins (L_p_) is easily accessible *via* a condensation reaction between the arylaldehyde and two and a half equivalents of 2,4-dimethylpyrrole, followed by oxidation by *p*-chloranil (Loudet and Burgess, 2007). The π-extended dipyrrins (L_π_) were obtained by Knoevenagel condensation of the plain dipyrrins at the methyl groups in *alpha* to the pyrrolic nitrogen with 2-napthalencarbaldehyde, catalysed by acetic acid (Tungulin et al., 2019). By mixing one equivalent of π-extended dipyrrin and one equivalent of a plain dipyrrin with zinc diacetate (Zn(OAc)_2_) at room temperature, the corresponding new heteroleptic bis(dipyrrinato) Zn^II^ complexes (L_p_ZnL_π_) were obtained with a yield of up to 40%, with chemical structures shown in Figure 1. Column chromatography is needed in order to separate the desired complexes from the homoleptic complexes (Zn(L_p_)_2_ and Zn(L_π_)_2_) that are also formed in the reaction (Figure 1). The homoleptic complexes **2a**, **2b**, and **3a** were already known, (respectively: (Sakamoto et al., 2016; Tsuchiya et al., 2016; Tungulin et al., 2019) while the other homoleptic complexes are presented here for the first time, to the best of our knowledge. We expect them to have a distorted tetrahedral geometry as other bis(dipyrrinato) Zn^II^ complexes (Kusaka et al., 2012; Tsuchiya et al., 2014; Zhang et al., 2018; Zhang et al., 2019).

### Photophysical Properties

**FIGURE 2:**
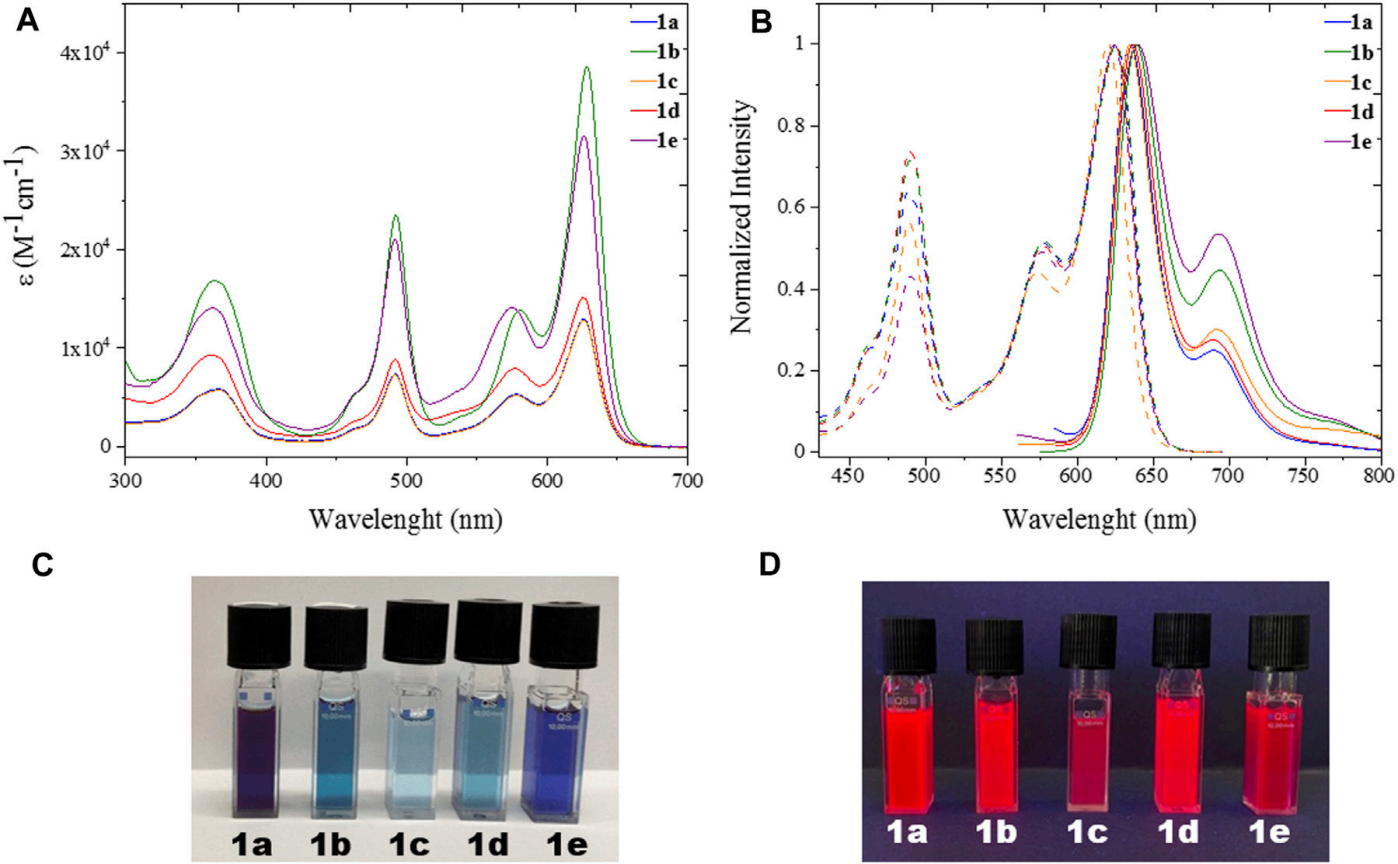
UV **(A)** Uv/Vis absorption spectra with molar absorptivity coefficient (Ɛ) and **(B)** excitation and emission spectra of heteroleptic complexes **1a-e** (λ_exc_ = 570 nm) in spectroscopic DMSO; **(C)** Pictures of **1a-e** in dimethyl sulfoxide solution under ambient (top) and **(D)** UV (bottom) light.

**FIGURE 3:**
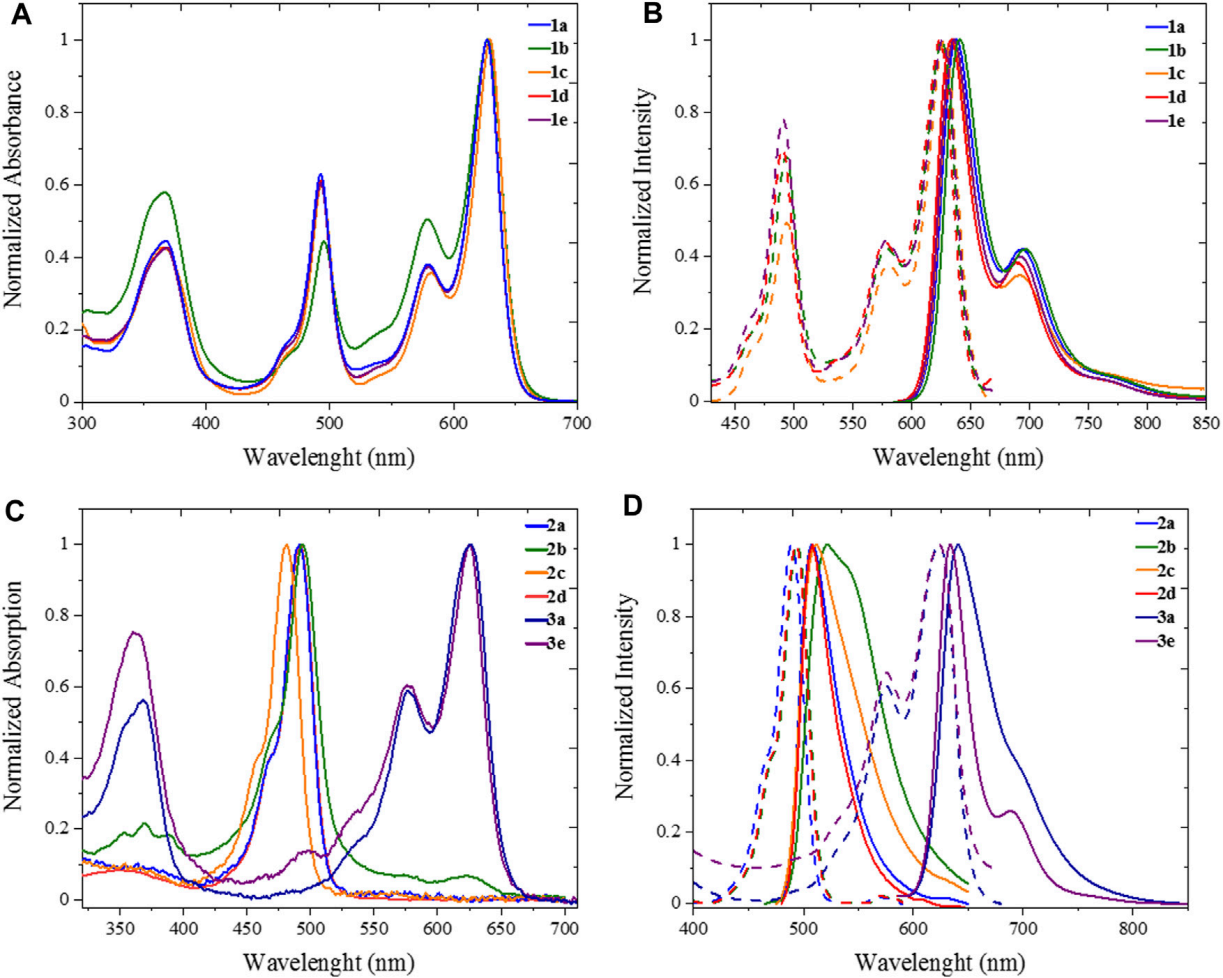
UV Photophysics in spectroscopic toluene for heteroleptic complexes **1a-e** (top) and homoleptic complexes **2a-d**, **3a** and **3e** (bottom). **(A)** Uv/Vis absorption and **(B)** excitation (dashed plot, λ_em_ = 710 nm) emission (solid plot, λ_exc_ = 570) spectra of **1a-e**. **(C)** Uv/Vis absorption and **(D)** excitation (dashed plot, λ_em_ = 600 nm for **2a-d**, λ_em_ = 700 nm for **3a**, **3e**) emission (solid plot, λ_exc_ = 470 nm for **2a-d**, λ_exc_ = 570 nm for **3a**, **3e**).

All five heteroleptic complexes **1a–e** have an intense blue colour in solution (Figure 2). Their spectroscopic properties were investigated in a nonpolar solvent, such as toluene (PhMe), and in a polar aprotic solvent, such as dimethyl sulfoxide (DMSO), which will be used for the preparation of the biological assays. In order to understand their photophysical properties, their relative homoleptic complexes were also characterised (see Figure 3 and Supplementary Table S1 in ESI). The UV-vis absorbance spectra of complexes **1a–e** have shared features, as shown in Figures 2, 3.

From the absorption spectra, we identify three main electronic transition bands in the heteroleptic complexes **1a–e** (Table 1). The broad band at high energy centred at ca. 360 nm is attributed to the electronic transitions localised on the naphthyl vinyl moieties of the π-extended dipyrrins, as they are absent in the plain dipyrrins. It is worth to notice that, in complex **1b**, the characteristic structured band of the anthracenyl moiety is not visible as it is hidden by the aforementioned naphthyl vinyl absorption. This is not the case for the homoleptic complex **2b**, in which spectrum the vibronic structure of the anthracene absorption is clearly visible. The other two main bands are in the visible region, and their profile is reminiscent of the absorption of the dipyrrin ligands. Between these two bands, the one at highest energy presents a shoulder at 465 nm and a relative maximum at ca. 490 nm (e.g.: ε (**1e**) = 2.2 10^4^ cm^−1^ M^−1^).

**TABLE 1:** Photophysical properties of heteroleptic Zn^II^ complexes **1a-e**.

Complex	λ_abs_^[a]^[nm] (Ɛ [10^4^ M^−1^cm^−1^])	λ_em_ [nm]	Δν [cm^−1^] (*Pseudo* Δν [10^3^ cm^−1^])	Φ ^[c]^	τ [d] [ns]	*k*_r_ [10^7^ s^−1^]	*k*_nr_ [10^7^ s^−1^]
**1a**	366 (0.54)	635^[a]^	0.20 (4.62) ^[a]^	0.44^[a]^	3.1^[a]^	14.2^[a]^	18.1^[a]^
	491 (0.70)	638^[b]^	0.30 (4.61) ^[b]^	0.71^[b]^	3.5^[b]^	20.3^[b]^	8.3^[b]^
	627 (1.24)						
**1b**	364 (1.73)	639^[a]^	0.27 (4.63) ^[a]^	0.37^[a]^	3.6^[a]^	10.3^[a]^	17.3^[a]^
	493 (2.4)	641^[b]^	0.21 (4.51) ^[b]^	0.55^[b]^	4.2^[b]^	13.1^[b]^	10.7^[b]^
	628 (3.89)						
**1c**	366 (0.57)	634^[a]^	0.20 (4.59) ^[a]^	0.05^[a]^	3.0^[a]^	1.8^[a]^	31.5^[a]^
	491 (0.72)	635^[b]^	0.17 (4.42) ^[b]^	0.18^[b]^	4.0^[b]^	4.5^[b]^	20.5^[b]^
	626 (1.28)						
**1d**	360 (0.95)	636^[a]^	0.27 (4.62) ^[a]^	0.46^[a]^	2.6^[a]^	17.0^[a]^	20.0^[a]^
	492 (0.90)	634^[b]^	0.32 (4.56) ^[b]^	0.63	3.8^[b]^	16.6^[b]^	9.7^[b]^
	625 (1.53)						
**1e**	361 (1.54)	640^[a]^	0.32 (4.66) ^[a]^	0.38^[a]^	3.2^[a]^	11.9^[a]^	19.4^[a]^
	493 (2.2)	636^[b]^	0.20 (4.53)^[b]^	0.45^[b]^	3.9^[b]^	11.5^[b]^	14.1^[b]^
	627 (3.28)						

^[a]^ Measured in DMSO and ^[b]^ in toluene. ^[c]^ Quantum yields were determined by the relative method, using cresyl violet in methanol as reference (Φ = 0.54).(Brouwer, 2011) ^[d]^ Exciting with a NanoLED source at 570 nm.

**FIGURE 4:**
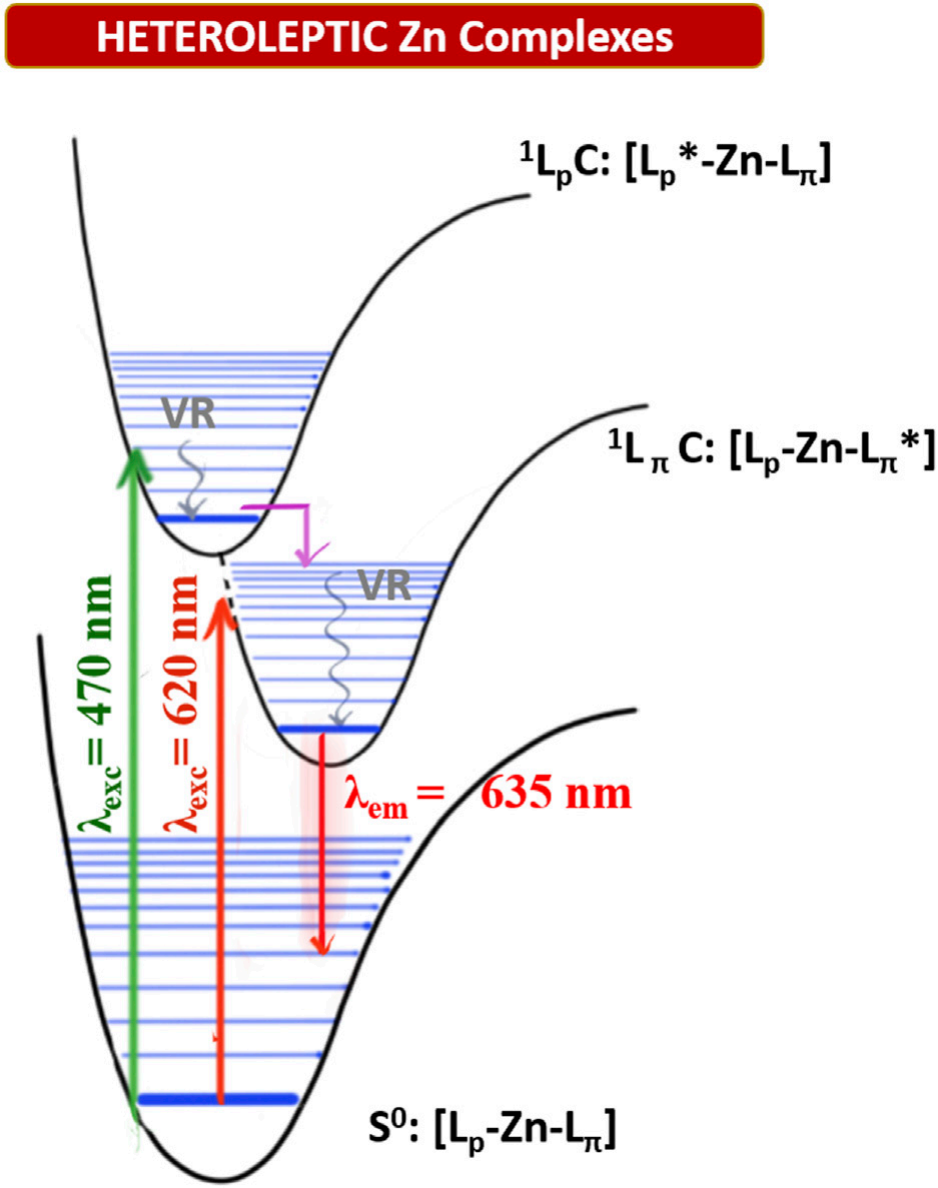
Qualitative Jablonski-diagram for the involved photophysical processes in heteroleptic Zn^II^ complexes. (VR: Vibrational radiation; ^1^L_p_C: singlet excited state centered on the plain dipyrrin; ^1^L_p_C: singlet excited state centered on the π-extended dipyrrin. Pink arrow: rapid interligand nonradiative transition).

This absorption is attributed to the singlet ligand centred (^1^LC) π→π* transition localised on the plain dipyrrin (^1^L_p_C in Figure 4). At longer wavelengths, a very intense absorption at ca. 620 nm (e.g.: ε (**1e**) = 3.3 10^4^ cm^−1^ M^−1^) is present with a shoulder at ca. 575 nm, which is assigned to the π→π* transition and its vibronic coupling localised on the π-expanded dipyrrin (^1^L_π_C in Figure 4). The Zn^II^ centre is a d^10^ metal, and it is not involved in the transitions. Furthermore, it is reasonable to expect that the dipyrrinato ligands are almost orthogonal to each other with a weak if not absent exciton coupling (Telfer et al., 2011; Trinh et al., 2014). Each complex shows fluorescence in the far-red region (emission centred at 635 nm) and a lower intensity shoulder in the near-infrared region up to 800 nm. The Φ values were measured by the relative method in two solvents: dimethyl sulfoxide (E_T_^N^: 0.44) and toluene (E_T_^N^: 0.099) (Reichardt, 1994). In a nonpolar solvent such as toluene, the zinc bis(dipyrrinato) complexes have the highest Φ, with values ranging from 18% for complex **1c** to 70% for complex **1a**. The difference in Φ among complexes **1a–e** has to be ascribed to the distinct aryl group in *meso*-position of the plain dipyrrins. It has been previously proven that the aryl group rotates in respect to the plain of the dipyrrin, allowing nonradiative deactivation unless bulky substituents impede this rotation (I.V. Sazanovich, 2004). In addition to that, the planarity of the chelating dipyrrin might change upon functionalisation, which also influences the rigidity and, therefore, the radiative transitions of the systems (Tungulin et al., 2019). The π-extended dipyrrin is the same for the heteroleptic compounds, besides complex **1e**, which possess a hydroxyl group in position 4 to the 2,6-dimethylphenyl substituent, which is in *para* to the dipyrrin. The presence of this extra hydroxyl group in **1e** with respect to **1d** might induce additional nonradiative processes since Φ in **1e** is slightly lower than the one in **1d** (Φ = 45% and Φ = 63%, respectively). The consistently lower emission efficiency in **1c** is related mainly to the increased rotational freedom of the aryl group in the *meso*-position of the plain dipyrrin. Furthermore, the electron-donating groups such as methoxy and hydroxyl groups induce an additional quenching effect. The homoleptic derivatives are emissive only in toluene (see Supplementary Table S1), although their Φ is much lower than their heteroleptic counterparts (e.g. Φ (**2a**): 18%). By comparing the emissions in a polar aprotic solvent such as DMSO, it is possible to assert that the emission energies are not affected by changing the polarity of the medium. In fact, LC transitions are not influenced by different polarities. Heteroleptic zinc bis(dipyrrinato) complexes are not symmetric in their ground and excited states. Therefore, the non-emissive symmetry breaking charge transfer state (SBCT) is not present, which is favoured in the case of homoleptic complexes instead (cf. Figure 4 and Supplementary Figure S5). The intensity of the emission of **1a–e** in DMSO, although reduced in comparison to the values obtained in PhMe, is still very strong with Φ of ca. 40%, except for complex **1c** (Φ = 5%). These values are incredibly appealing for far-red/near-IR emitters, especially because by lowering the emission energies, the nonradiative deactivation paths are much more probable to occur.

The fluorescence decays are monoexponential, and the lifetimes (τ) are close to 3 ns (in DMSO) and 4 ns (in PhMe), with minor differences among the complexes. Radiative rate constants (*k*_r_) are comparable among the heteroleptic Zn^II^ complexes and are higher than the nonradiative ones (*k*_nr_) in PhMe and lower in DMSO (except for complex **1c**). Excitation spectra of the investigated complexes show a precise comparison with their relative absorption spectra, meaning that the far-red emission centred on the π-extended dipyrrinato moiety also occurs upon excitation of the plain dipyrrinato moiety (Figures 2B, 3B, and Supplementary Figure S1). Thus, the excited state ^1^L_p_C undergoes a rapid interligand nonradiative transition to populate the lower-lying ^1^L_π_C (Figure 4). Therefore, upon excitation at shorter wavelength (470 nm), the detected emission is at lower energies (emission maximum at ca. 635 nm). This effect prompts a *pseudo*-Stokes shift of more than 4,600 cm^−1^ (Table 1). As the quenching of the plain dipyrrin is total, Nishihara and coworkers suggested a 100% efficient energy transfer from the donor L_p_ to the acceptor L_π_. (Kusaka et al., 2012; Sakamoto et al., 2016). Advanced studies are necessary in order to elucidate the photophysical pathways of these heteroleptic complexes and, currently, we are investigating the involved nonradiative processes by means of transient absorption spectroscopy, which are beyond the scope of the present work.

Further experiments were done by measuring the fluorescence lifetimes of the heteroleptic complexes **1a–e**, using three different excitation wavelengths (455, 570, and 625 nm). For each complex, the obtained decays show identical fittings independently from the excitation energy used (Supplementary Figure S1).

### Confocal Laser Microscopy

The emission colors of these new heteroleptic Zn^II^ bis(dipyrrinato) complexes, as well as their high quantum yields also in polar solvents and their large *pseudo*-Stokes Shifts are appealing properties for their exploitation in bioimaging. Before evaluating their biocompatibility, we analysed their stability in different aqueous environments. The UV/vis absorption spectra of the complexes **1a–e** were recorded in Dulbecco Modified Eagle Medium (DMEM) and in deionized water (Supplementary Figure S3), using the same concentrations adopted for the confocal laser microscopy, in order to evaluate these the new dyes in media close to the cellular environment. The absorption profiles are stable. The same is true also in aqueous solutions at pH 3.3 and 5.0 (Supplementary Figure S4). These conditions were chosen based on the typical pH gradient in endocytic compartments of cells and DMEM is tipically used as cellular medium for cell culture applications. Emission profiles of the compounds in DMEM overlay very well with those measured in organic solvents, while there is a bathochromic shift in water, where the emission maxima are at about 670 nm (Supplementary Table S1). Quantum yields of the compounds in aqueous media reflect the extremely large polarity (E_T_^N^of H_2_O: 1.00), since the values are up to 3.1% in DMEM and up to 1.7% in water. Furthermore, it should be noted that water causes an additional quenching effect due to hydrogen-bond-assisted nonradiative deactivation (Maillard et al., 2021).

The stability of these complexes was tested at increasing temperatures (Supplementary Figure S6. The emission of the complexes is only slightly reduced when going from 20 to 50°C, and this can be ascribed to the increasing collisions with solvent molecules followed by an increase of nonradiative deactivation processes. Thus, our far-red emissive bis(dipyrrinato) zinc complexes are stable in an aqueous solution at different pH values and temperatures. In order to test their biocompatibility in living cells, cell viability and cellular uptake were determined in four different cell types, including primary somatic cells such as human dermal fibroblasts (NHDF), a mouse cell line from embryonic fibroblasts (NIH3T3), and two human cancer cell lines (HeLa, and MCF7). To test the viability, MTT assays were performed by treating 10^4^ cells of the respective cell type with different concentrations of the complexes **1a–e** for 72 h at 37°C. For all complexes, the LD_50_ values were >20 μM, showing high biocompatibility (Supplementary Figure S7). On the contrary, MTT assays of the single dipyrrin ligand showed a decreased viability already at concentrations lower than 7 µM (Supplementary Figure S8). For all these results, we assume high stability of these heteroleptic complexes in the cellular environments.

**FIGURE 5:**
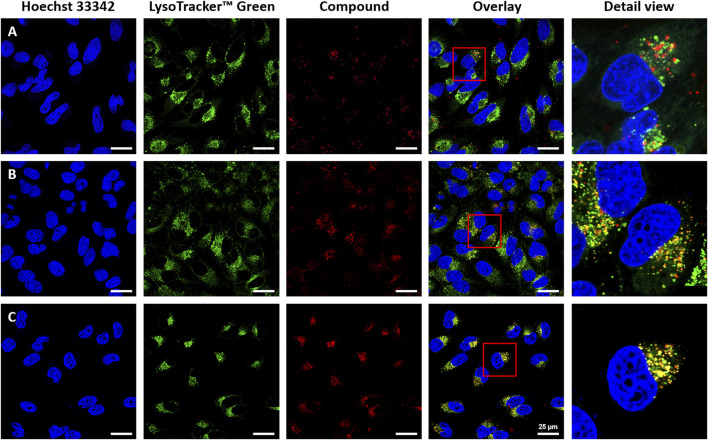
Cellular uptake of heteroleptic bis(dipyrrinato) zinc complexes in HeLa cells (**(A)**: **1a**, **(B)**: **1d**, **(C)**: **1e**). For co-staining of nuclei and endosomes, cells were treated with Hoechst 33,342 (λ_exc_ = 405nm, λ_em_ = 414–462 nm), Lysotracker™ Green (λ_exc_ = 488 nm, λ_em_ 494–545 nm); and Hoechst 33,342, compounds **1a**, **1d**, **1e** (λ_exc_ = 630 nm, λ_em_ = 640–750 nm). Intracellular accumulation of the complexes was detected with fluorescence confocal microscopy using a Leica Stellaris 5 with a white light laser. The overlay is the merged image of the single-channel fluorescence images. Scale bars: 25 μm.

**FIGURE 6:**
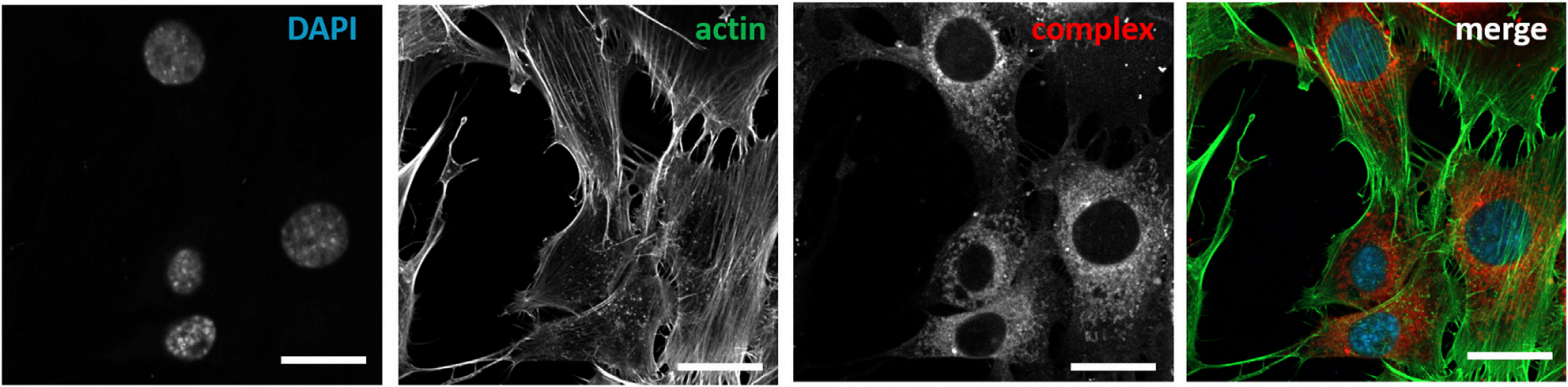
Post-fixation immunochemical staining of nuclei and actin cytoskeleton of NIH3T3 cells treated with compound **1d**. Cells were treated with DAPI (λ_exc_ = 405nm, λ_em_ = 410–470 nm), Actin (λ_exc_ = 488 nm, λ_em_ 490–535 nm); compound **1d** (λ_exc_ = 640 nm, λ_em_ = 656–700 nm).

Since all the complexes showed only negligible toxicity when used to treat the different cell lines, a concentration of 20 µM was chosen for the cellular uptake experiments and the live-cell fluorescent imaging (Figure 5, 6 and Supplementary Figures S11–17). With the best performing complexes **1d** and **1e** we tested their cellular uptake also at different concentrations, such as 1, 10, and 20 µM (Supplementary Figures S9, S10) It was assumed that due to their hydroxyl groups, a higher water solubility was achieved with complexes **1c**, **1d**, and **1e**. As expected, due to the low Φ of **1c**, this complex is hardly detectable. In contrast, complexes **1d** and **1e** display improved cellular uptake with respect to DMSO controls by virtue of their higher solubility in aqueous solution. Complexes **1a** and **1b** showed decreased cellular uptake. All complexes were taken up by endocytosis at the respective concentrations, leading to an accumulation in the endosomal/lysosomal compartment, which was proven by the counterstaining with Lysotracker Green^TM^ (Figure 5) (Canton and Battaglia, 2012; Kolmel et al., 2012).

As a further analysis, confocal live-cell fluorescence microscopy was performed with Mitotracker™ Green. However, no counterstaining with mitochondrial markers was observed (Supplementary Figures S12–14). Figures 5A–C shows the counterstaining experiments in HeLa cells with Lysotracker Green with Pearson coefficients of 0.85 for **1e**, 0.60 for **1d**, and 0.24 for **1a** (Bolte and Cordelières, 2006). To further test the suitability of these heteroleptic Zn^II^ bis(dipyrrinato) complexes for bioimaging applications, the correlation of incubation time with signal intensity in live-cell imaging and the stability after fixation of cells were also investigated. While differences in the obtained signal intensity could be detected when incubating cells for a period of 0.5, 1.5, and 6 h (Supplementary Figure S17, compound **1e**), the complexes showed no decrease in fluorescence intensity after fixation (Figure 6). This proves the versatility of these complexes, as they can be used in live-cell imaging and in fixed-cell experiments, e.g. for immunocytochemistry. Moreover, given the photochemical properties of the compounds and the significant *pseudo*-Stokes shift, the excitation of complexes was possible at multiple wavelengths, allowing for the simultaneous excitation of two fluorophores at 488 nm and the detection of their emission at different wavelengths.

## Conclusion

In the search of promising far-red-emitting fluorophores for bioimaging, we designed and synthesized five new heteroleptic Zn^II^ bis(dipyrrinato) complexes. Their relative homoleptic derivatives were prepared for comparison. We investigated their luminescence in two different solvents: the nonpolar toluene and the polar and water-miscible dimethylsulfoxide. In contrast to the homoleptic derivatives, the heteroleptic complexes feature high emission also in polar aprotic solvent, such as DMSO. We confirmed that emission comes only from the singlet excited state that is centered on the π-extended dipyrrin that has the lowest energy gap. Therefore, those heteroleptic complexes emit in the far-red to NIR region, which is highly desirable for biological investigations. Our heteroleptic Zn^II^ bis(dipyrrinato) are stable in aqueous solutions and at different pH. They presented an endosomal uptake with high cell biocompatibility in four different cell types. Thanks to their large *pseudo*-Stokes shift, these complexes can be excited at multiple wavelengths. Moreover, we demonstrated that they can be used also in fixed-cell experiments. All in one, those results envision heteroleptic Zn^II^ bis(dipyrrinato) complexes as successful fluorophores and motivates further development for exciting application in fluorescence imaging and beyond.

## Data Availability

The original contributions presented in the study are included in the article/Supplementary Material, further inquiries can be directed to the corresponding author.
