# Supplementary material for: Intriguing Heteroleptic ZnII bis(dipyrrinato) Emitters in the Far-Red Region With Large Pseudo-Stokes Shift for Bioimaging
Source: Front Chem. 2021 Sep 23;9:754420. doi: 10.3389/fchem.2021.754420 (PMC8495118; doi:10.3389/fchem.2021.754420)
Supplement: Supplementary file 1 [file DataSheet1.pdf]

Supplementary Material

Table of contents

1. Materials and methods 2
1.1 General 2
1.2 Photophysics 2
1.3 Cytotoxicity Assay 2
1.4 Cell Culture, confocal laser microscopy and co-staining experiments 3
1.5 Cross-linking fixative procedure for fixed-cells confocal laser microscopy 3
2. Synthetic procedures 4
2.1 Synthesis of plain dipyrrin **4a-d** 4
2.2 Synthesis of π-expanded dipyrrin **5a-e** 5
2.3 Synthesis of Heteroleptic Bis(dipyrrinato) Zn^{II} complexes **1a-e** 6
2.4 Synthesis of Homoleptic bis (dipyrrinato) Zn^{II} complexes **2a-d**, **3a**, **3e** 8
3. Supplementary Figures 10
3.1 Photophysical properties of heteroleptic complexes **1a-e** in DMSO 10
3.2 Fluorescence lifetimes of complexes **1a-e** in DMSO 11
3.3 Photophysical properties of heteroleptic complexes **1a-e** in DMEM and water. 12
3.4 Photophysical properties of homoleptic complexes **2a-d**, **3a**, **3e** in toluene 13
3.5 Emission spectra of heteroleptic complexes **1a-e** at different temperature 14
3.6 Cell Cytotoxicity 15
3.7 Confocal laser microscopy 16
3.7.1 Cellular Uptake of complex **1d** at different concentrations in NIH3T3 and MCF7 cells. 16
3.7.2 Cellular Uptake of complex **1e** at different concentrations in NIH3T3 and MCF7 cells. 17
3.7.3 Cellular Uptake of complex **1a-e** at 20 μM in NIH3T3 and MCF7 cells. 18
3.7.4 Intracellular co-localization experiments of complex **1a-e** in HeLa cells. 19
3.7.5 Intracellular co-localization experiments of complex **1a-e** in NHDF cells. 20
3.7.6 Intracellular co-localization experiments of complex **1a-e** in NIH3T3 cells. 21
3.7.7 Intracellular co-localization experiments of complex **1a-e** in HeLa cells. 22
3.7.8 Intracellular co-localization experiments of complex **1a-e** in NIH3T3 cells. 23
3.7.9 Cellular uptake over time of complexes **1a-e** in NIH3T3 and MCF7 cells. 24
4. ^{1}H-NMR and ^{13}C-NMR spectra of complexes. 25

1. Materials and methods

1.1 General

Reagents and solvents employed were commercially acquired from Sigma Aldrich, ABCR, Thermo Fisher Scientific or Carl Roth and, unless otherwise stated, used without previous purification. Dry solvents were provided from an automatic solvent purification system (SPS) model 800 manuals of ^{M}BRAUN. DMEM (Dulbecco Modified Eagle Medium) cell culture medium, DPBS (Dulbecco´s Phosphate Buffered Saline), HyClone™ BGS (bovine growth serum supplemented calf), FCS (fetal calf serum), Penicillin/Streptomycin (Gibco), Trypsin-EDTA 0.25% (Gibco) were used for cell culture. CellTiter 96 Non-Radioactive Cell Proliferation Assay (Promega), Triton X-100 (Serva), MitoTracker™ Green FM, LysoTracker™ Green DND-26, Hoechst 33342, BioTracker™ 405 Blue Mitochondria Dye and LysoTracker®Blue DND-22 were used for cell imaging.

^{1}H-NMR and ^{13}C-NMR data were recorded at room temperature in $CDCl_3$ by a Bruker 400 spectrometer. Mass spectral determination were performed with a MALDI-ToF System employing a reflection tuning mode and using 6-aza-2-thiothymine (ATT) as matrix. The microwave-assisted reaction was conducted in a Biotage ® Initiator+ Microwave System with Robot Sixty.

1.2 Photophysics

The absorption spectra were recorded with a PerkinElmer Lambda 750 double-beam Uv/Vis-NIR spectrometer. Fluorescence was measured with a Jobin-Yvon Fluoromax 4 fluorimeter with a step width of 1 mm. All measurements were performed in quartz cuvettes with septum from Hellma and, where not explicated, conducted at 20 °C through the use of an Advanced Ac200 Immersion Circulators Thermostats-Thermo Fisher Scientific. Solvents for spectroscopy were supplied by Merck (Uvasol). Photoluminescence quantum yields were determined utilizing as standard Cresyl Violet in spectroscopic methanol (Φ in MeOH, 22 °C = 0.54). Lifetime measurements were performed by time-correlated single-photon counting method (TCSPC) with a DeltaTime kit for DeltaDiode source on Fluoromax systems, including DeltaHuB and DeltaDiode controller. The light-sources were NanoLED sources (455nm, 570 nm and 625 nm).

1.3 Cytotoxicity Assay

The cytotoxicity was assessed by using the CellTiter 96 Non-Radioactive Cell Proliferation Assay (Promega) according to producer´s instructions. It is based on the intracellular reduction of the yellow 3-(4,5-dimethylthiazol-2-yl)-2,5-di-phenyltetrazolium bromide (MTT) to a blue formazan product. Briefly, 1 x 10^4 cells of respective cells type (HeLa, NHDF, NIH3T3 and MCF7) were seeded in each well of a 96-well plate and incubated with DMEM, supplemented with 10% of FCS and 1% of Penicillin/Streptomycin (37 °C, 5% of CO_2 atmosphere) for 24 h. Then, the cell culture medium was removed and fresh DMEM containing new compounds was added to the cells which were incubated for 72 h. In order to have an accurate spectrum of the results, the colorimetric assay was performed on HeLa cells incubated with ligands **4a-d, 5a, 5e** at different concentration (1, 2.5, 5, 7.5 µM). Subsequently, viability levels of HeLa and NHDF cells were evaluated by incubating with complexes **1a-e** at different concentration (1, 2.5, 5, 7.5, 10 and 20 µM) for 72 h. Considering that the concentration used for the microscopy is 20 µM and expecting a comparable trend, NIH3T3 and MCF7 were treated with complexes

1a-e only at 20 µM. One negative control was realized by incubating cells in DMEM + DMSO 20 µM. After adding 5 µl of Triton-X100 (20% in ultra-pure and sterile water ddH_2O) to the positive control, 15 µl of MTT-solution was added in each well and the cells were incubated for additional 3h before adding 100 µL of a stop-solution mix. The absorption was measured at 570 nm by using a Spectramax ID3 (HeLa and NHDF) or a BioTek Synergy LX (NIH3T3 and MCF7) multi-well reader after an incubation period of 24 h, to allow the solubilization of the formazan product. Each experimental well and controls were prepared in triplicates.

1.4 Cell Culture, confocal laser microscopy and co-staining experiments

Confocal live-cell fluorescence microscopy was performed by seeding 1.5 x 10^4 cells on each chamber of an µ-slide 8-well (ibidi®) (for HeLa and NHDF) and on 35mm-glass bottom dishes (ibidi®) (for NIH3T3 and MCF7) followed by an incubation period of 24 h in DMEM supplemented with 10% of FCS and 1% of Penicillin/Streptomycin (37°C, 5% of CO_2 atmosphere). Subsequently, medium was removed and fresh DMEM, containing **1a-e** (20 µM, o.4% v/v), was added to the cell culture which was incubated for additional 24 h. Afterwards, MitoTracker™ Green (125 nM) or LysoTracker™ Green (50 nM) was added and, after 30 min, cells were washed three times with DPBS and fresh medium with Hoechst 33342 (2 µg/ml) was included.

Due to the possibility of exciting the fluorophores at different excitation wavelength, in order to prove the feasibility of different imaging conditions, BioTracker™ 405 Blue Mitochondria Dye (125 nM) and LysoTracker®Blue DND-22 (50 nM) were also employed. Co-staining experiments were performed following the same procedure as above mentioned, with the exception of the Hoechst 33342 which, in this case, was not added. The microscopy was performed by using, for HeLa and NHDF cell type, a confocal Leica Stellaris microscope with a HC PL Apo CS2 63x oil objective (N.A.= 1.4) and, for NIH3T3 and MCF7 cell type, a confocal Zeiss LSM800 microscope with a C-Apo 40x water immersion objective (N.A.=1.2). Acquisition parameters (e.g. excitation/emission wavelengths) were adjusted according to the specific investigation as indicated in the figure captions. The Pearson correlation coefficient (PCC) was calculated by using the JACoP plugin for ImageJ program. (Bolte and Cordelières, 2006)

1.5 Cross-linking fixative procedure for fixed-cells confocal laser microscopy

For the fixation experiments, DMEM was removed after cell culture (containing complex **1d**) and replaced with paraformaldehyde (4% in PBS) for 15min. Cells were then washed three times with PBS+ 0.1% of Triton X-100 for 5min. Subsequently, cells were incubated with a solution of phalloidin coupled to AlexaFluor488 (Invitrogen, dilution 1:200) and DAPI (4,6-diamidino-2-phenylindole) (Roth, dilution 1:1000) in PBS+ 1% of BSA for one hour, to stain the actin cytoskeleton and the nuclei, respectively. Finally, cells were washed three times with PBS, kept in buffer and imaged.

2 Synthetic procedures

2.1 Synthesis of plain dipyrrin 4a-d

General procedure: The synthesis of plain dipyrrins, a condensation of 2,4-dimethyl-1*H*-pyrrole and the appropriate aromatic aldehyde, was performed following the procedure described in literature (I.V. Sazanovich) with some improvements.

Synthesis of 4a: 2,4-dimethyl-1H-pyrrole (4.918 g, 51.69 mmol, 2.5 equiv.) and 2,4,6-trimethylbenzaldehyde (3.064 g, 20.67 mmol, 1 equiv.) were solved in dichloromethane (250 ml), then was added hydrochloric acid 0.2 M (60 ml). The reaction was carried out at room temperature overnight. Subsequently, 2,3,5,6-Tetrachlorocyclohexa-2,5-diene-1,4-dione (p-Chloranil) (5.592 g, 22.74 mmol, 1.10 equiv.) was added and the reaction mixture was left stirring at room temperature for 8 hours. The organic layers were then washed with saturated NaCl aqueous solution three times, collected and dried over $MgSO_4$ anhydrous, filtered and evaporated under vacuum. The obtained crude product was purified *via* column chromatography, packed with alumina, using dichloromethane as eluent. The target compound was isolated as a dark-orange powder (5.200 g, 16.33 mmol, 79 % yield); Rf= 0.6 in CH_2Cl_2.

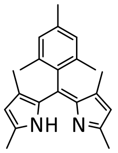

4a

^{1}H-NMR (400 MHz, $CDCl_3$) δ/ppm: 6.91 (s, 2H), 5.86 (s, 2 H), 2.35 (s, 6H), 2.33 (s, 3H), 2.11 (s, 6H), 1.31 (s, 6H).^{13}C-NMR (400 MHz, $CDCl_3$) δ/ppm:151.23, 139.77, 137.81, 137.65, 135.81, 135.60, 134.10, 128.68, 128.36, 119.11, 21.31, 19.69, 16.21, 13.72.

Synthesis of 4b: the reaction of 2,4-dimethyl-1H-pyrrole (3.460 g, 36.37 mmol, 2.5 equiv.) with 9-anthracenecarboxaldehyde (3.00 g, 14.55 mmol, 1 equiv.) was performed in dichloromethane (250 ml) and hydrochloric acid 0.2 M (60 ml). The reaction was carried out at room temperature for 24 hours. Subsequently, 2,3,5,6-Tetrachlorocyclohexa-2,5-diene-1,4-dione (p-Chloranil) (3.934 g, 16.00 mmol, 1.10 equiv.) was added and the reaction mixture was left stirring for 24 hours. The organic layers were then washed with saturated NaCl aqueous solution three times, collected and dried over $MgSO_4$ anhydrous, filtered and evaporated under vacuum. The obtained crude product was purified *via* column chromatography, packed with alumina, using dichloromethane as eluent. The target compound was isolated as a black powder (4.286 g, 11.38 mmol, 78 % yield). Rf= 0.5 in CH_2Cl_2.

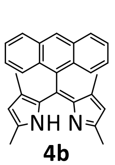

4b

^{1}H-NMR (400 MHz, $CDCl_3$) δ/ppm: 8.54 (s,1H), 8.00 (m, 4H), 7.45 (t, J=8.3 Hz, 2H), 7.37 (t, J= 8.1 Hz, 2H), 5.78 (s, 2 H), 2.42 (s, 6H), 0.51 (s, 6H).^{13}C-NMR (400 MHz, $CDCl_3$) δ/ppm:151.73, 140.10, 137.37, 131.78, 130.59, 130.35, 128.17, 127.35, 126.92, 125.83., 125.65, 125.44, 119.48, 108.18, 16.15, 13.60.

Synthesis of 4c: 2,4-dimethyl-1H-pyrrole (3.127 g, 32.86 mmol, 2.5 equiv.) and 4-hydroxy-3-methoxy-benzaldehyde (2.00 g, 13.15 mmol, 1 equiv.) were solved in dichloromethane (250 ml), then was added hydrochloric acid 0.2 M (60 ml) to the reaction mixture which was carried out at room temperature for 24 hours. Then 2,3,5,6-Tetrachlorocyclohexa-2,5-diene-1,4-dione (p-Chloranil) (2.737 g, 11.13 mmol, 1.10 equiv.) was added and the reaction mixture was left

stirring for 24 hours. The organic layers were then washed with saturated NaCl aqueous solution three times, collected and dried over $MgSO_4$ anhydrous, filtered and evaporated under vacuum. The obtained crude product was purified *via* column chromatography, packed with alumina, using a gradient of dichloromethane/methanol as eluent. The target compound was isolated as a black powder (1.560 g, 48.39 mmol, 37 % yield); Rf= 0.3 in CH_2Cl_2.

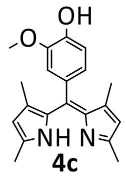

4c

^{1}H-NMR (400 MHz, $CDCl_3$) δ/ppm: 7.33 (m, 2 H), 7.22 (dd, J= 4.8 Hz, 1.7 Hz, 1H), 5.79 (s, 2 H), 3.5 (s, 3H), 2.24 (s, 6H), 1.20 (s, 6H);^{13}C-NMR (400 MHz, $CDCl_3$) δ/ppm:136.34, 134.39, 133.54, 130.06, 129.36, 129.54, 128.92, 128.42, 54.68, 26.87,14.32.

Synthesis of 4d: the reaction of 2,4-dimethyl-1H-pyrrole (2.376 g, 24.97 mmol, 2.5 equiv.) with 4-hydroxy-2,6-dimethylbenzaldehyde (1.500 g, 9.988 mmol, 1 equiv.) was performed in a mixture of dichloromethane (150 ml)/methanol (50 ml) and hydrochloric acid 0.2 M (30 ml). The reaction was carried out at room temperature for 24 hours, and after the addition of 2,3,5,6-Tetrachlorocyclohexa-2,5-diene-1,4-dione (p-Chloranil) (2.947 g, 11.99 mmol, 1.10 equiv.), it was left stirring for 24 hours. The organic layers were then washed with saturated NaCl aqueous solution three times, collected and dried over $MgSO_4$ anhydrous, filtered and evaporated under vacuum. The obtained crude product was purified *via* column chromatography, packed with alumina, using dichloromethane as eluent. The target compound was isolated as a dark-green powder (2.000 g, 6.242 mmol, 62 % yield); Rf= 0.4 in CH_2Cl_2.

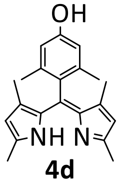

4d

^{1}H-NMR (400 MHz, $CDCl_3$) δ/ppm: 6.57 (s, 2H), 5.86 (s, 2 H), 5.30 (s, 1H), 2.34 (s, 6H), 2.07 (s, 6H), 1.34 (s, 6H).^{13}C-NMR (400 MHz, $CDCl_3$) δ/ppm:155.30, 151.37,139.76, 137.69, 137.25, 135.75, 129.56, 119.11, 114.64, 53.45, 19.74, 16.06, 13.71.

2.2 Synthesis of π-expanded dipyrrin 5a-e

General Procedure: The π-conjugation in dipyrromethene ligands was achieved through Knoevenagel condensation. (Dalessandro et al., 2017)

Synthesis of 5a: according to the general procedure (Tungulin et al., 2019) compound **4a** (3.130 g, 9.829 mmol, 1equiv.) and naphthalene-2-carbaldehyde (9.210 g, 58.97 mmol, 6 equiv.) were dissolved in 200 ml of dry toluene. After that, 5 ml of piperidine and 5 ml of glacial acetic acid were added dropwise to the reaction mixture, refluxed for 24 hours, using an air-cooled condenser under argon atmosphere. After this time, the organic layer was washed with saturated NaCl aqueous solution, the aqueous layers were recombined and extracted with CH_2Cl_2. The organic phases were collected and dried over $MgSO_4$.The mixture was filtered and the solvent was evaporated under reduced pressure. The crude residue was purified *via* flash column chromatography on alumina gel (gradient of cyclohexane/dichloromethane), giving the product as a purple solid (1.7g, 2.539 mmol, 27% yield). Rf=0.8 (CH_2Cl_2).

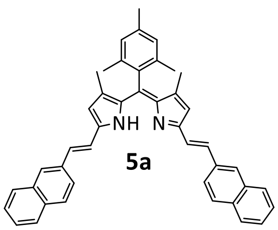

5a

^{1}H-NMR (400 MHz, $CDCl_3$) δ/ppm: 7.86-7.75 (m, 8 H), 7.65 (dd, J= 7.9, 1.6 Hz, 2H), 7.45-7.36 (m, 6H), 7.22 (d, J= 2.0 Hz, 2H), 6.91 (s, 2H) 6.37 (s, 2H), 2.31 (s, 3H),2.12 (s, 6 H), 1.37 (s, 6H).^{13}C-NMR (400 MHz, $CDCl_3$) δ/ppm: 150.71, 140.46, 138.88, 135.89, 134.74, 133.73, 133.28, 131.82, 128.71, 128.52, 128.24, 127.73, 127.44, 126.45, 126.17, 123.47, 121.31, 119.12, 26.94, 21.26, 19.79, 13.87.

Synthesis of 5e: following the general procedure (Mani et al., 2017), in a round bottom microwave glass vial (16mm OD X 83 mm Long) compound **4d** (1.00 g, 3.121 mmol, 1equiv.) and naphthalene-2-carbaldehyde (2.924 g, 18.72 mmol, 6 equiv.) were dissolved in 4 ml of ethanol and then 1 ml of piperidine and 1 ml of glacial acetic acid were added dropwise. The solution was refluxed for 2 hours at 100 °C under microwave irradiation. After cooling the system, the organic layer was washed with saturated NaCl aqueous solution and the aqueous layers were recombined and extracted with CH_2Cl_2. The organic phases were collected and dried over $MgSO_4$.The mixture was filtered and the solvent was evaporated under reduced pressure. The crude residue was purified *via* flash automatic column chromatography on silica gel (gradient of dichloromethane/methanol) giving the product as a purple solid (500 mg, 0.8379 mmol, 27% yield); Rf=0.4 (CH_2Cl_2).

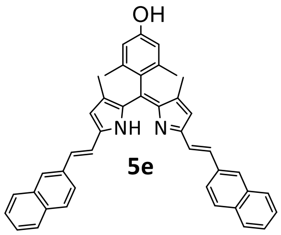

5e

^{1}H-NMR (400 MHz, $CDCl_3$) δ/ppm: 8.07 (s, 2 H), 7.99 (s, 2 H), 7.96-7.91 (d, J= 1.2 Hz,4 H), 7.88-7.83 (dt, J=7.1 Hz, 4 H), 7.57 (s, 2H), 7.53-7.50 (h, J=3.7 Hz, 2 H), 7.48 (s, 2 H), 7.44 (s, 2 H), 6.63 (s, 2H), 5.75 (s, 1H), 2.01 (s, 6H), 1.41 (s, 6H). ^{13}C-NMR (400 MHz, $CDCl_3$) δ/ppm: 151.10, 140.99, 140.22, 137.34, 137.04, 136.21, 135.26, 134.66, 133.76, 128.76, 128.56, 128.09, 127.75, 127.67, 127.16, 126.68, 126.52, 123.48, 121.26, 119.48, 55.31, 24.52, 14.33.

2.3 Synthesis of Heteroleptic Bis(dipyrrinato) Zn^{II} complexes 1a-e

General procedure: two different kind of functionalized dipyrrin (1 equiv. each) were dissolved in 50 ml of dichloromethane and 25 ml of methanol. Then, zinc diacetate (1.5 equiv.) was added and the reaction was left stirring overnight at room temperature. The solvent was removed under reduced pressure and the crude residue was purified by flash chromatography. **Synthesis of 1a:** The reaction of **4a** (369.0 mg, 1.738 mmol, 1equiv.) and **5a** (551.4 mg, 0.9270 mmol, 1 equiv.) with zinc diacetate (318.9 mg, 1.783 mmol, 1.5 equiv.) was performed in a 2:1 mixture of CH_2Cl_2/MeOH. Column chromatography packed with alumina neutral was used for purification (gradient of cyclohexane/dichloromethane as eluent mixture) and gave **1a** as a blue powder (200 mg, 0.204 mmol, 18 % yield).

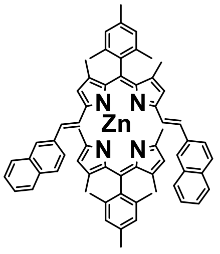

1a

^{1}H-NMR (400 MHz, $CDCl_3$) δ/ppm: 7.80-7.72 (m, 6H), 7.63 (s, 1H), 7.61 (s, 1H), 7.58 (s, 2H), 7.44 (ddd, J=7.0, 2.6, 1.5 Hz, 6H), 7.30 (dd, J=8.6, 8.1 Hz, 2H), 7.18 (s, 1H), 7.13 (s, 1H), 7.00 (s, 2H), 6.82 (s, 2H), 6.67 (s, 2H), 2.40 (s, 3H), 2.31 (s, 3H), 2.19 (s, 12H), 1.91 (s, 6H), 1.44 (s, 12H). ^{13}C-NMR (400 MHz, $CDCl_3$) δ/ppm: 150.85, 140.59, 139.02, 138.07, 137.61, 136.02, 134.87, 133.86, 133.41, 131.96, 128.85, 128.65, 128.37, 127.86, 127.57, 126.58, 126.31, 123.60, 121.45, 119.26, 27.07, 21.39, 19.92, 14.00. HRMS (MALDI) m/z calcd for $C_{66}H_{62}N_4Zn^+$: 974.42659; found: 974.4455. Elemental analysis for $C_{66}H_{62}N_4Zn$: C 81.17, H 6.40, N 5.74; found C 81.77, H 6.50, N 5.90.

Synthesis of 1b: The reaction of **4b** (500.0 mg, 1.382 mmol, 1equiv.) and **5a** (656.9 mg, 1.382 mmol, 1 equiv.) with zinc diacetate (365.5 mg, 1.992 mmol, 1.5 equiv.) was performed in a 2:1 mixture of CH_2Cl_2/MeOH. Column chromatography packed with neutral alumina was used for purification (gradient of cyclohexane/dichloromethane as eluent mixture) and gave **1b** as a blue-violet powder (315 mg, 0.304 mmol, 23 % yield).

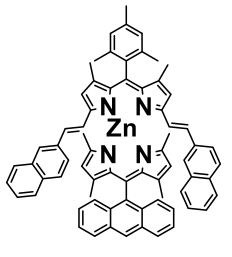

1b

^{1}H-NMR (400 MHz, $CDCl_3$) δ/ppm: 8.50 (s, 1H), 7.88 (t, J= 8.2 Hz, 4 H), 7.67-7.63 (m, 4 H), 7.62-7.58 (m, 2H), 7.43-7.30 (m, 13 H), 7.05-6.98 (m, 4 H), 6.75 (s, 2H) 6.26 (ddd, J= 7.9, 6.5, 1.3 Hz,2 H), 5.97 (s, 2H) 2.42 (s, 3H), 2.26 (d, J=9.7, 2H), 1.49 (s, 6H), 0.64 (s, 6H). ^{13}C-NMR (400 MHz, $CDCl_3$) δ/ppm: 157.72, 156.32, 144.66, 144.17, 142.97, 141.86, 138.74, 136.44, 135.50, 134.14, 133.51, 132.90, 131.78, 131.17, 129.39, 128.95, 128.55, 127.92, 127.37, 126.78, 126.41, 126.30, 126.19, 125.48, 124.55, 123.33, 121.28, 117.95, 27.37, 21.73, 20.12, 17.09, 15.71, 15.51. HRMS (MALDI) m/z calcd for $C_{71}H_{60}N_4Zn^+$: 1032.41094; found: 11032.9434. Elemental analysis for $C_{71}H_{60}N_4Zn•H_2O$: C 81.01, H 5.94, N 5.32; found: C 81.17, H 5.03, N 5.44.

Synthesis of 1c: The reaction of **4c** (135.5 mg, 0.420 mmol, 1equiv.) and **5a** (250 mg, 0.420 mmol, 1 equiv.) with zinc diacetate (154.2 mg, 0.841 mmol, 1.5 equiv.) was performed in a 2:1 mixture of CH_2Cl_2/MeOH. Column chromatography packed with alumina was used for purification (gradient of dichloromethane/methanol as eluent mixture) and gave **1c** as a blue-violet powder (150 mg, 0.153 mmol, 36 % yield).

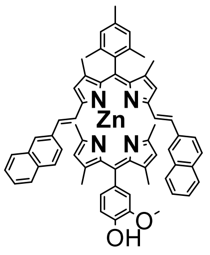

1c

^{1}H-NMR (400 MHz, $CDCl_3$) δ/ppm: 7.72-7.61 (m, 5H), 7.52 (d, J=8.6 Hz, 3 H), 7.50-7.43 (m, 2H), 7.39-7.31 (m, 5H), 7.25 (dd, J=8.6, 1.7 Hz, 3H), 7.15 (s, 1H), 7.11 (s, 2H), 7.02 (s, 2H), 6.97 (s, 1H), 6.75 (s, 2H), 6.69 (s, 2H), 2.25 (s, 3H), 1.83 (s, 6H), 1.47 (s, 3H), 1.43 (s, 6H), 1.35 (s, 9H).). ^{13}C-NMR (400 MHz, $CDCl_3$) δ/ppm: 156.52, 144.07, 138.57, 137.92, 136.36, 135.17, 133.72, 133.18, 133.03, 128.92, 128.21, 128.09, 127.77, 127.61, 126.61, 126.26, 125.97, 123.98, 122.75, 118.59, 27.07, 21.39, 19.81, 15.60. HRMS (MALDI) m/z calcd for $C_{64}H_{58}N_4O_2Zn^+$:978.38512; found:978.3020. Elemental Analysis for $C_{64}H_{58}N_4O_2Zn$ •2 H_2O: C 75.61, H 6.15, N 5.51 found: C 75.12, H 6.10, N 5.49.

Synthesis of 1d: The reaction of **4d** (150.0 mg, 0.4681 mmol, 1equiv.) and **5a** (167.1 mg, 0.281 mmol, 1 equiv.) with zinc diacetate (103.1 mg, 0.562 mmol, 1.5 equiv.) was performed in a 2:1 mixture of CH_2Cl_2/MeOH. Automatic column chromatography packed with silica gel was used for purification (gradient of dichloromethane/methanol as eluent mixture) and gave **1d** as a blue-violet powder (180.0 mg, 0.1836 mmol, 39 % yield).

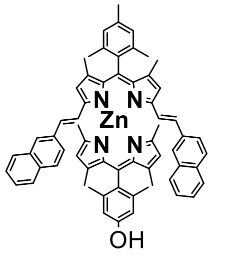

1d

^{1}H-NMR (400 MHz, $CDCl_3$) δ/ppm: 7.77-7.69 (m, 4 H), 7.59 (d, J=8.6 Hz, 2H), 7.55 (s, 2H), 7.44-7.38 (m, 4H), 7.24 (d, J= 1.9 Hz, 2 H), 7.19 (s, 1H), 7.15 (s, 1H), 7.07 (s, 1H), 7.03 (s, 1H), 6.98 (s, 2H), 6.65 (s, 2H), 6.48 (s, 2H), 6.04 (s, 2H), 2.38 (s, 3H), 2.16 (s, 12 H), 1.84 (s, 6H), 1.46 (s, 6H), 1.40 (s, 6H). ^{13}C-NMR (400 MHz): 151.17, 140.91, 139.33, 138.39, 137.94, 136.34, 135.19, 134.18, 133.73, 132.28, 129.17, 128.98, 128.69, 126.90, 126.63, 123.92, 121.77, 119.57, 53.88, 27.39, 21.71, 20.24, 14.32. HRMS (MALDI) m/z calcd for $C_{65}H_{60}N_4OZn^+$: 974.2659; found: 974.4455. Elemental Analysis for $C_{65}H_{60}N_4OZn$ • CH_2Cl_2 calcd: C 74.54, H 5.88, N 5.27; found: C 74.69, H 5.90, N 5.42.

Synthesis of 1e: The reaction of **4d** (300.0 mg, 0936 mmol, 1equiv.) and **5e** (391.1 mg, 0.655 mmol, 1 equiv.) with zinc diacetate (257.7 mg, 1.404 mmol, 1.5 equiv.) was performed in a 2:1 mixture of CH_2Cl_2/MeOH. Automatic column chromatography packed with silica was used for purification (gradient of cyclohexane/dichloromethane) and gave **1e** as a blue-violet powder (98.60 mg, 0.099 mmol, 40 % yield).

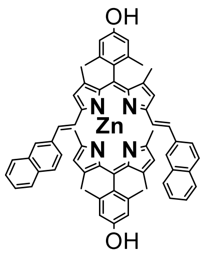

1e

^{1}H-NMR (400 MHz, $CDCl_3$) δ/ppm: 7.83-7.72 (m, 12H), 7.62 (dd, J= 8.0, 1.6 Hz), 7.44-7.31 (m, 7H), 7.23 (s, 2H), 6.89 (s, 2H), 6.34 (s, 2H), 2.29 (s, 6H), 2.09 (s, 9 H), 1.35 (s, 12H), 1.18 (s, 3 H). ^{13}C-NMR (400 MHz): 151.17, 140.91, 139.34, 138.39, 137.94, 136.34, 135.49, 135.19, 134.18, 133.73, 129.17, 128.97, 126.69, 128.18, 127.89, 126.90, 126.93, 123.92, 121.77, 119.57, 30.17, 21.71, 20.23, 15.91, 14.32. HRMS (MALDI) m/z calcd for $C_{64}H_{58}N_4O_2Zn^+$: 978.3851; found: 978.5610. Elemental Analysis for $C_{64}H_{58}N_4O_2Zn \cdot 2H_2O$: C 75.61, H 6.15, N 5.51; found: C 76.02, H 6.20, N 5.70.

2.4 Synthesis of Homoleptic bis (dipyrrinato) Zn^{II} complexes 2a-d, 3a, 3e

General procedure for 2a, 2b, 3a: the synthesis of complexes **2a**, **2b, 3a** was performed according to the procedure already present in literature (Sakamoto et al., 2016;Tsuchiya et al., 2016;Tungulin et al., 2019).

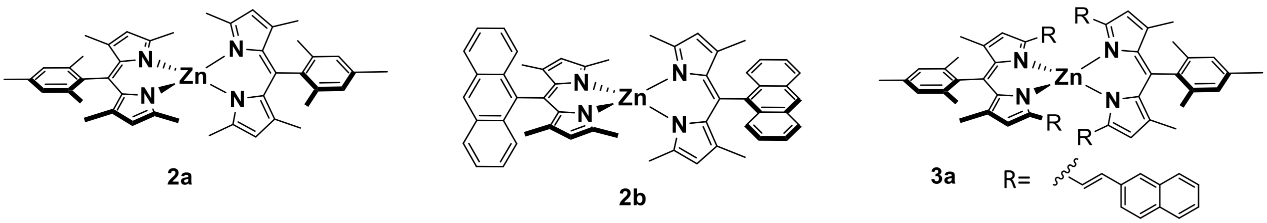

^{1}HNMR and ^{13}CNMR analysis matched with literature values: **2a and 3a** according to reference 5, and **2b** according to reference 3.

Synthesis of 2c: To a solution of **4c** (61.56 mg, 0.1635 mmol, 2equiv.) in dichloromethane (10 mL), zinc acetate (15.0 mg, 0.082 mmol, 1 equiv.) pre-solved in methanol (5ml) was added and stirring overnight to give **15** as a dark-green powder (40 mg, 0.048 mmol, 60 % yield).

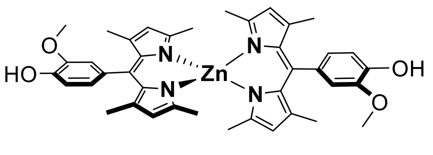

2c

^{1}H-NMR (400 MHz, $CDCl_3$) δ/ppm: 7.45 (m, 2 H), 7.35 (m, 4 H), 5.79 (s, 4 H), 3.51 (s, 3H), 3.32 (s, 3H), 2.12 (s, 12 H), 1.18 (s, 12H);^{13}C-NMR (400 MHz, $CDCl_3$) δ/ppm:155.76, 143.09, 143.01, 137.20, 136.08, 135.42, 134.39, 128.59, 119.41, 21.06, 19.10, 15.96, 14.67. HRMS (MALDI) m/z calcd for $C_{40}H_{42}N_4O_4Zn^+$: 706.24975 found: 705.94350 Elemental Analysis for $C_{40}H_{44}N_4O_5Zn \cdot 2\ H_2O$: C 64.56, H 6.23, N 7.53; found: C 64.80, H 6.41, N 7.64

Synthesis of 2d: To a solution of **4d** (34.93mg, 0.1090 mmol, 2equiv.) in dichloromethane (10 mL), zinc acetate (10.0 mg, 0.054 mmol, 1 equiv.) pre-solved in methanol (5ml) was added and stirring overnight to give **4d** as a dark-green powder (25.4 mg, 0.036 mmol, 66% yield).

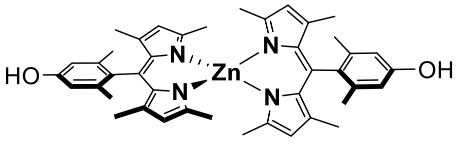

2d

^{1}H-NMR (400 MHz, $CDCl_3$) δ/ppm: 6.53 (s, 4H), 5.86 (s, 4H), 5.27 (s, 1H) 2.40 (s, 12H), 2.07 (s, 12H), 1.34 (s, 12H). ^{13}C-NMR (400 MHz): 155.28, 151.34, 139.73, 137.71, 135.75, 119.10, 114.60, 19.74, 16.06, 13.72. HRMS (MALDI) m/z calcd for $C_{42}H_{46}N_4O_2Zn^+$: 702.29122; found: 702.69228. Elemental analysis for $C_{42}H_{46}N_4O_2Zn$ •$2H_2O$ calcd: C 68.15, H 6.81, N 7.57; found: C 68.74, H 6.75, N 7.41.

Synthesis of 3e: To a solution of **5d** (65.05mg, 0.1090 mmol, 2equiv.) in dichloromethane (10 mL), zinc acetate (10.0 mg, 0.054 mmol, 1 equiv.) pre-solved in methanol (5ml) was added and stirring overnight to give **3e** as a dark-blue powder (50 mg, 0.039 mmol, 73% yield).

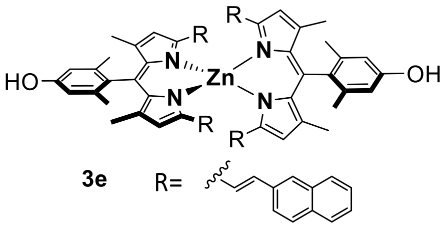

3e

^{1}H-NMR (400 MHz, $CDCl_3$) δ/ppm: 7.77-7.68 (dd, J= 1.09, 9H), 7.59 (d, J= 8.55, 4H), 7.54 (s, 4H), 7.44-7.39 (m, 8H), 7.32 (dd, J= 1.70, 8.65, 4H), 7.21(s, 2H), 7.17 (s, 2H), 7.09 (s, 2H), 7.05 (s, 1H), 6.82 (s, 4H), 6.76 (s, 4H), 1.89 (12 H), 1.52 (d, 12H). ^{13}C-NMR (400 MHz, $CDCl_3$) δ/ppm: 156.38, 144.39, 143.41, 138.69, 138.24, 136.68, 136.18, 135.48, 134.04, 133.49, 133.35, 129.24, 128.53, 128.41, 128.08, 127.92, 126.58, 126.29, 124.29, 123.07, 118.90, 21.70, 20.12, 15.91.HRMS (MALDI) m/z calcd for $C_{88}H_{70}N_4O_2Zn^+$: 1254.47902 found: 1254.0528: Elemental Analysis for $C_{88}H_{70}N_4O_2$•2 H_2O calcd: C79.89, H5.77, N 4.33; found: C 79.98, H 5.81, N 4.62.

3 Supplementary Figures

3.1 Photophysical properties of heteroleptic complexes 1a-e in DMSO

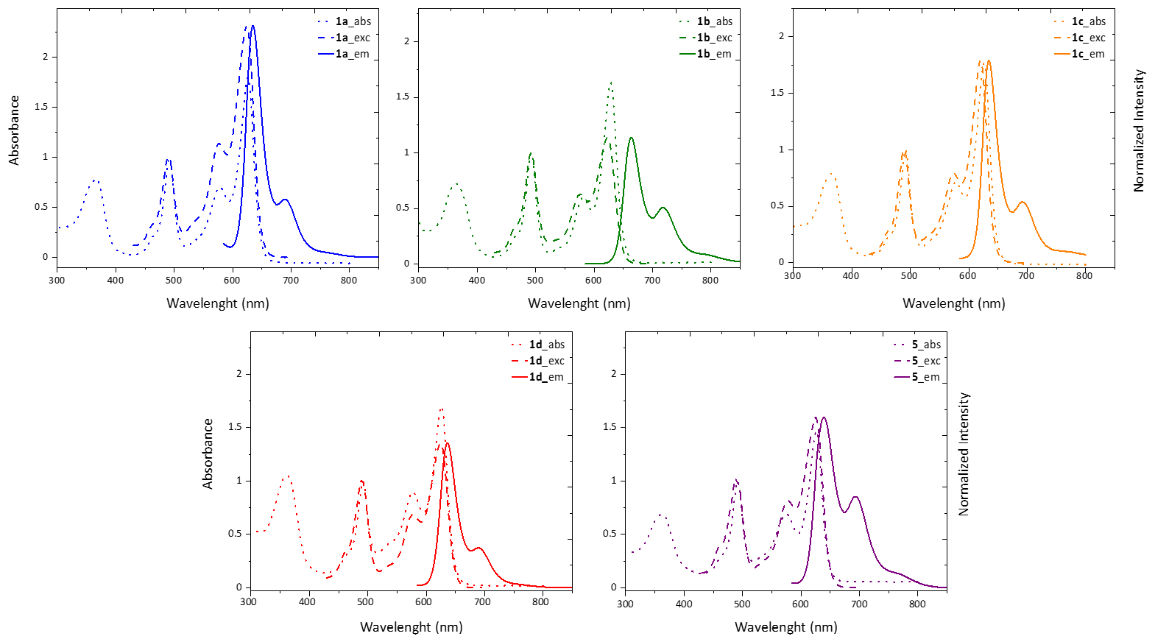

Figure **S1**: UV/Vis absorption (dotted plot), excitation (dashed plot, λ_{em} = 710 nm) and emission (solid plot, λ_{exc} = 570 nm) spectra of heteroleptic complexes **1a-e** in DMSO. Absorption and excitation values have been normalized to the (^{1}LC) π→π* transition localized on the plain dipyrrins (ca.490 nm). Emission values have been normalized to the maximum of the excitation spectra, which is attributed to the π→π* transition of the π-expanded dipyrrin.

3.2 Fluorescence lifetimes of complexes 1a-e in DMSO

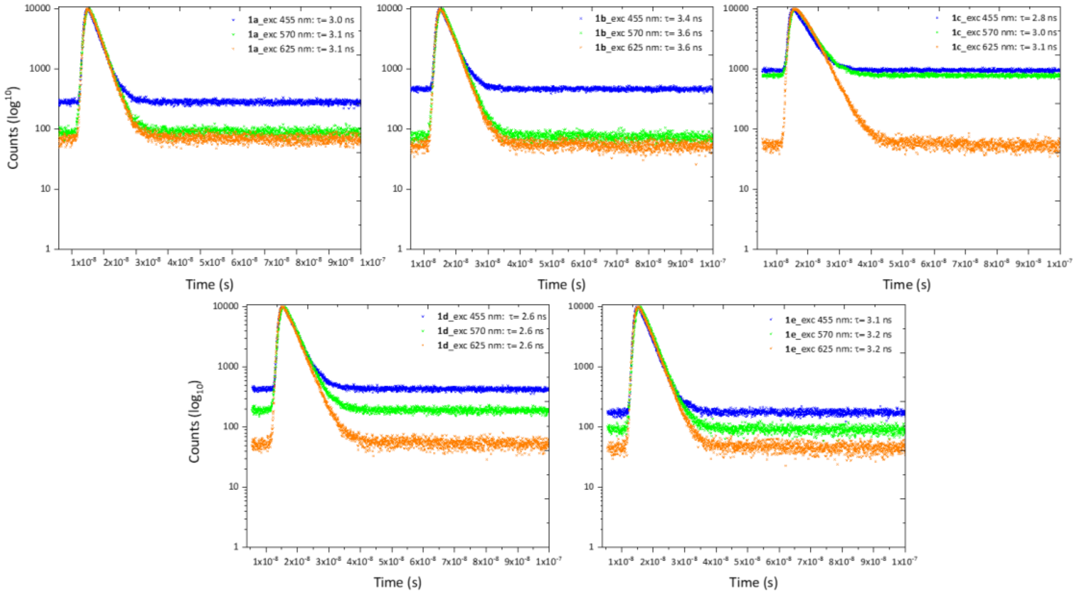

Figure **S2**: Fluorescence lifetimes of heteroleptic complexes **1a-e** in DMSO exciting with NanoLED source at three different excitation wavelengths (λ_{exc} 455, 570, 625 nm). Baselines appear different depending on the excitation laser wavelength, due to different signal to noise ratio. In fact, an enhanced noise is caused by scattering and other sources. Nevertheless, single exponential decay curves are independent of the excitation wavelength and are reported in Table 1 of the manuscript.

3.3 Photophysical properties of heteroleptic complexes 1a-e in DMEM and water.

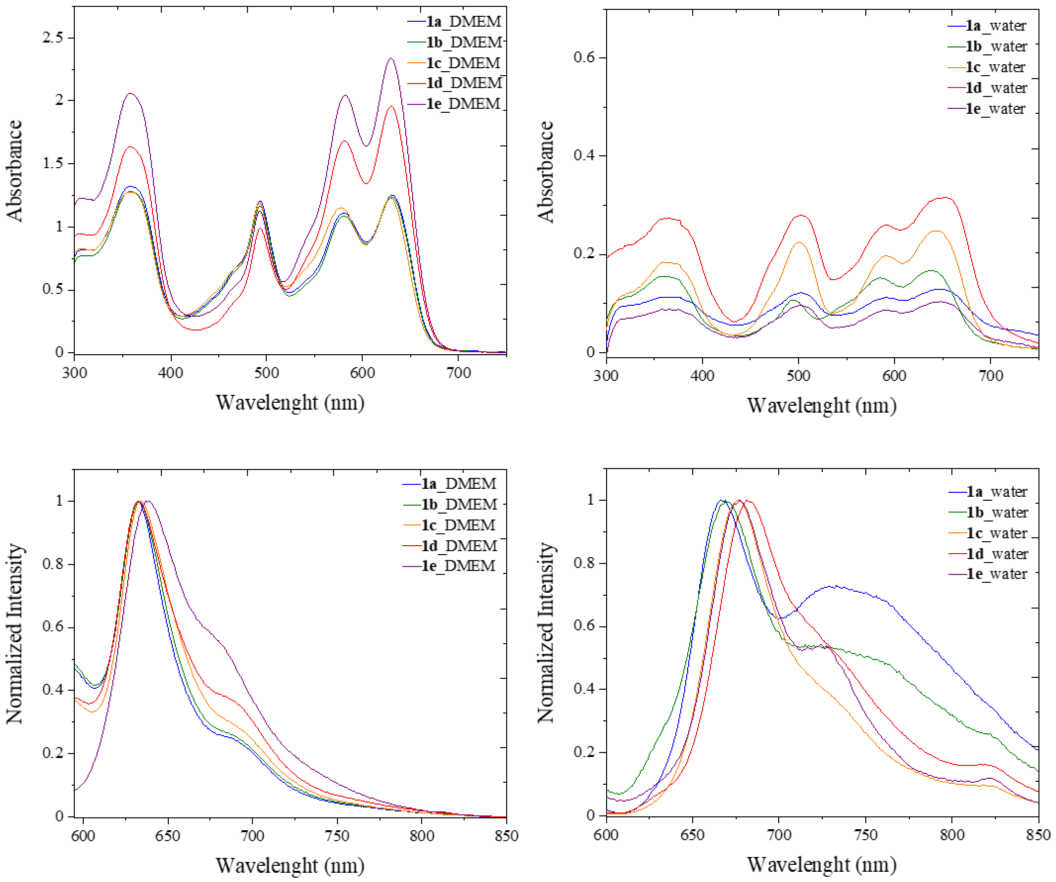

Figure S3: Uv/Vis absorption (top) and emission (bottom) spectra of complexes **1a-e** (20 µM) in DMEM (Dulbecco Modified Eagle Medium, supplemented with 10% of FCS) (left) and water (right). Emission spectra were recorded exciting at λ_{exc} = 570 nm. The broader band at lower energies in samples **1a** and **1b** is probably due to nanosized aggregates.

Table S1: Photophysical properties of complexes **1a-e** in [a] DMEM and [b] water.

Complex	λ_{em} *[a]* [nm]	Φ*[a]* (%)
**1a**	633 [a], 666 [b]	1.9 [a],0.8 [b]
**1b**	637 [a], 669 [b]	1.6 [a], 0.6 [b]
**1c**	635 [a], 676 [b]	1.0 [a], 0.6 [b]
**1d**	634 [a], 681[b]	2.0 [a], 1.7 [b]
**1e**	636 [a], 677 [b]	3.1 [a], 0.8 [b]

[a] Quantum yields were determined by the reference method, using as reference cresyl violet in methanol (Φ = 0.54).(Brouwer, 2011).

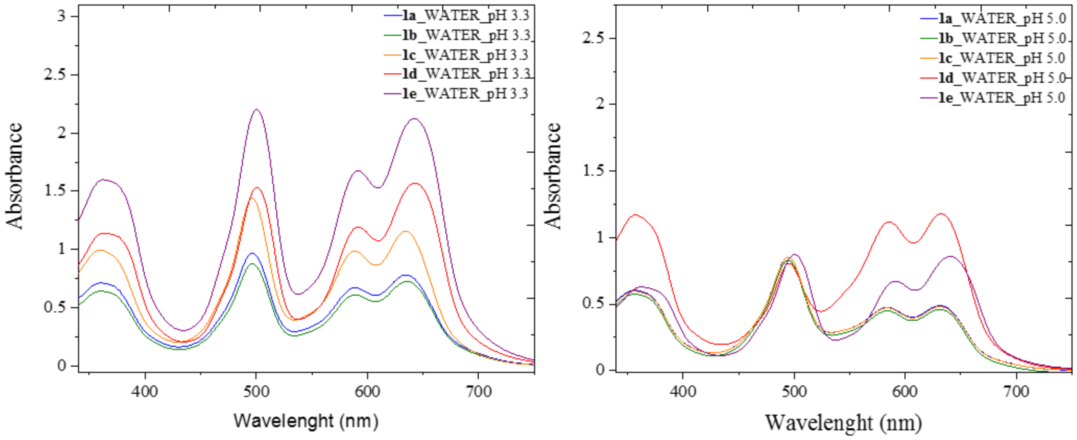

Figure S4: Uv/Vis absorption spectra of complexes **1a-e** (20 µM) in water at different pH (3.3 and 5.0). The pH was adjusted by using some drops of HCl.

3.4 Photophysical properties of homoleptic complexes 2a-d, 3a, 3e in toluene.

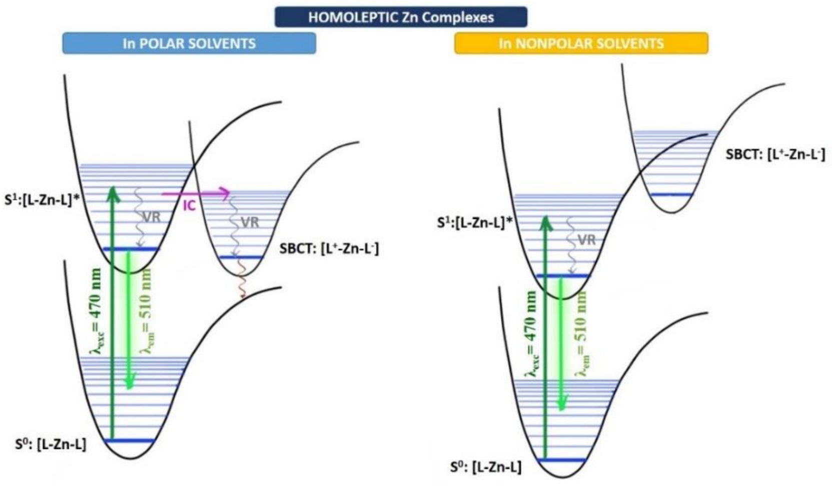

Figure **S5**: Qualitative Jablonski diagram for the involved photophysical processes in homoleptic complexes (**2a-d, 3a, 3e**) in polar and non-polar solvents.

Table **S2**: Photophysical properties of homoleptic complexes **2a-d, 3a, 3e** in toluene.

**Complex**	**λ_{abs} [nm]**	**λ_{em} [nm]**	**Φ [a, b]**	**τ [c, d] [ns]**	**k_r (10^7) [s^{-1}]**	**k_{nr} (10^7) [s^{-1}]**
**2a**	491	508	0.18[a]	2.5 [c]	5.7	25.5
**2b**	494	522	0.16[a]	2.3 [c]	4.9	26.2
**2c**	496	512	0.13[a]	2.2 [c]	3.9	27.3
**2d**	492	508	0.14[a]	2.8 [c]	4.4	26.8
**3a**	625	641	0.24[b]	3.9 [d]	7.6	23.7
**3e**	626	637	0.23[b]	4.2 [d]	7.3	23.9

[a] Quantum yields were determined by the reference method, using as reference $Ru(bpy)_3Cl_2$ in water (Φ = 0.028) and [b] cresyl violet in methanol (Φ = 0.54).(Brouwer, 2011) [c] Exciting with a NanoLED source at 455 nm for compounds **2a-d** and [d] by using NanoLED source at 570 nm for compounds **3a, 3e.**

3.5 Emission spectra of heteroleptic complexes 1a-e at different temperature

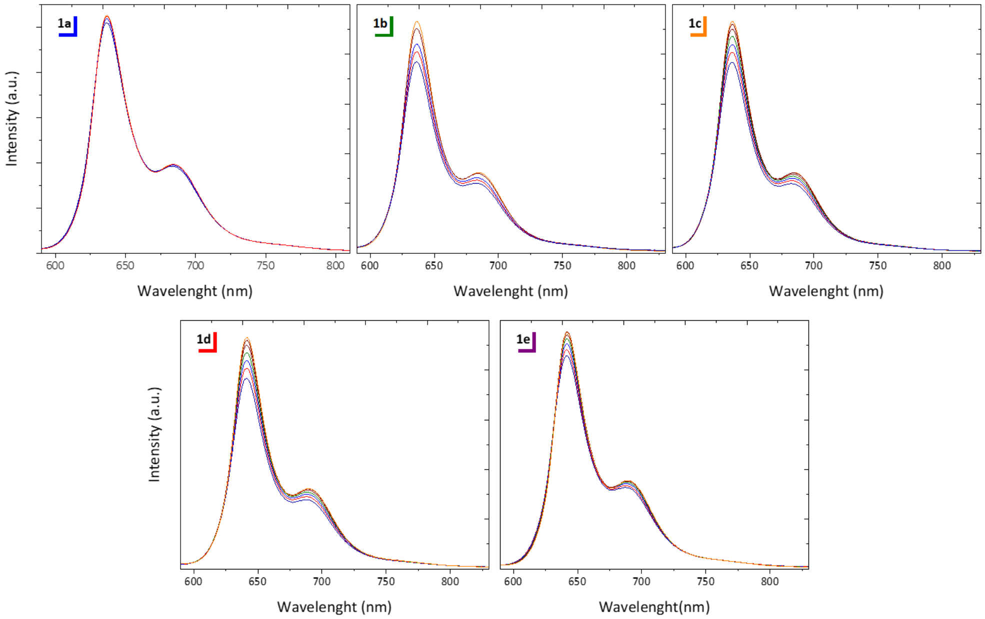

Figure **S6**: Emission spectra of heteroleptic complexes **1a-e** (λ_{exc} = 570 nm) recorded from 20°C to 50 °C (5°C of intervals in between).

3.6 Cell Cytotoxicity

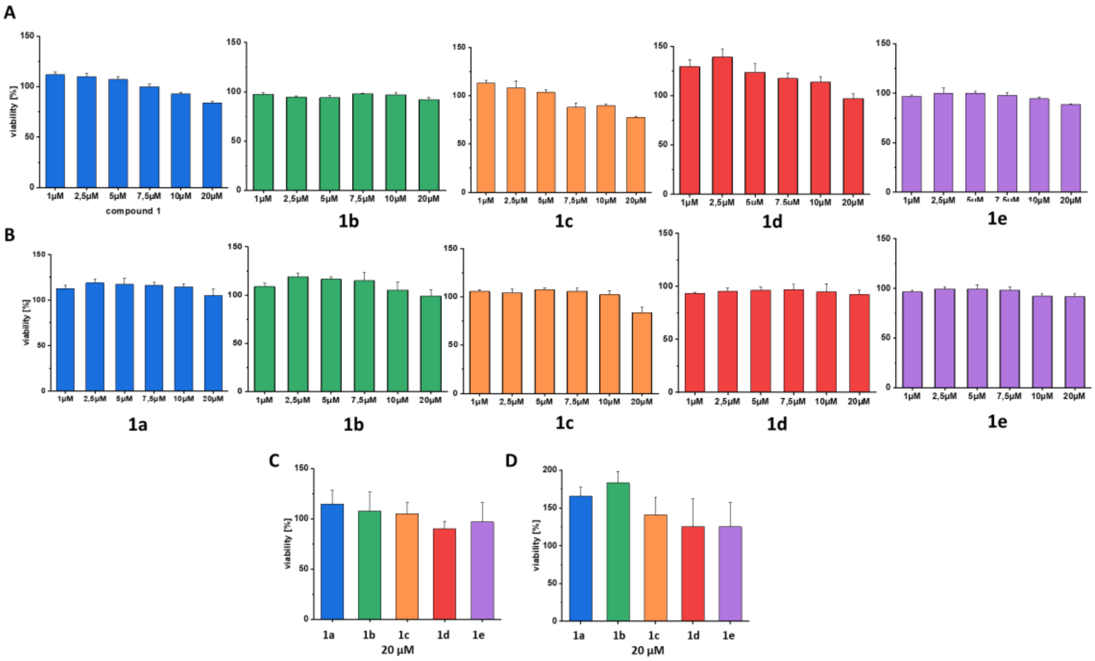

Figure **S7**: Potential cell viability (%) of (A) HeLa (B) NHDF (C) NIH3T3 and (D) MCF7 cells after 72 h of incubation with complexes **1a-e** (37 °C, 5 % of CO_2).

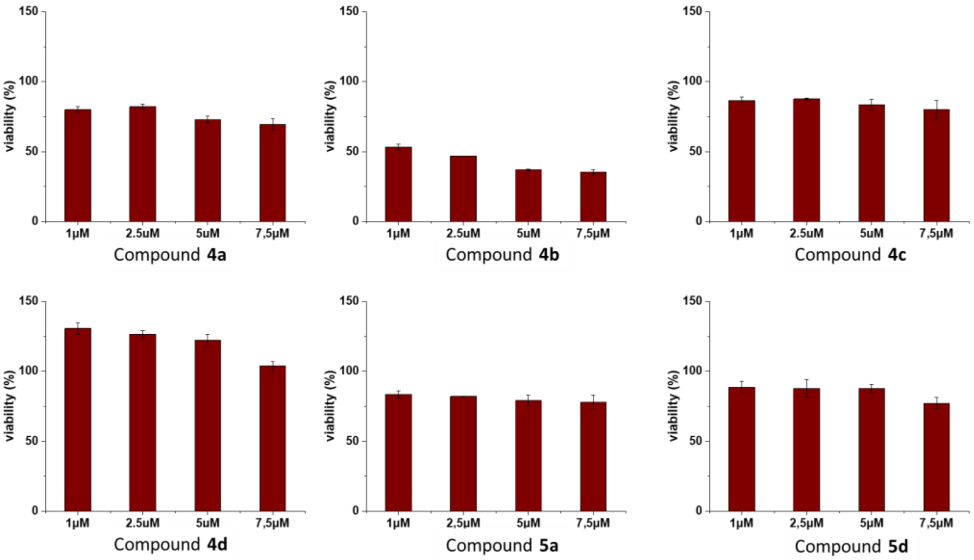

Figure **S8**: Potential cell viability (%) of HeLa cells after 72 h of incubation with ligands **4a-4d, 5a, 5d** (37 °C, 5 % of CO_2

3.7 Confocal laser microscopy

3.7.1 Cellular Uptake of complex 1d at different concentrations in NIH3T3 and MCF7 cells.

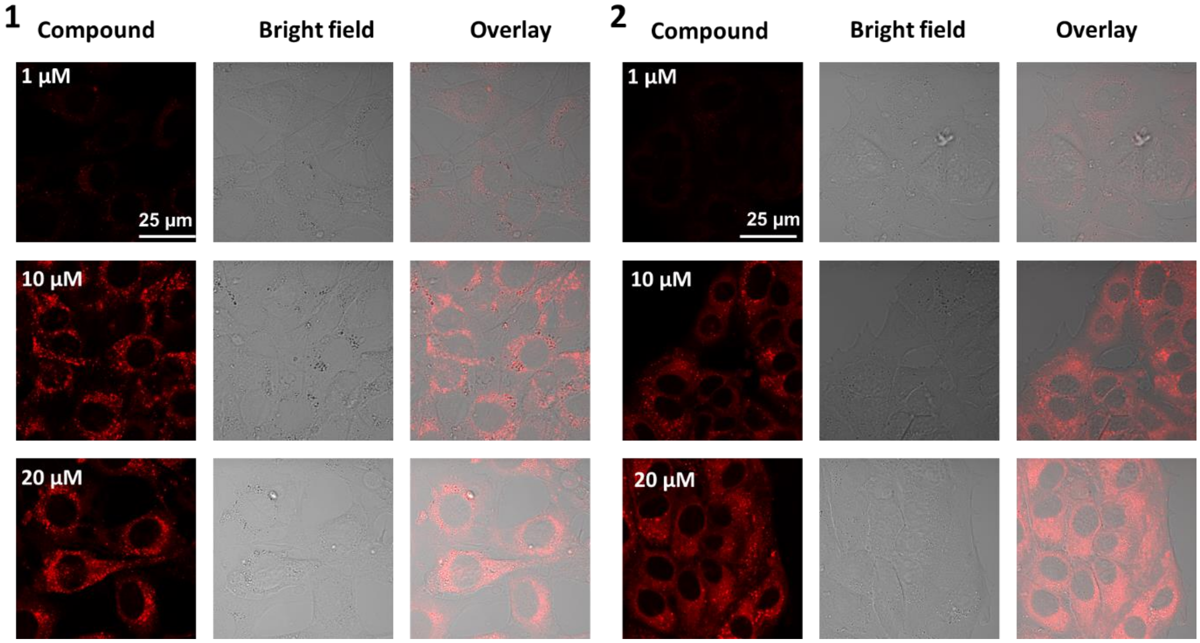

Figure S9: Cellular uptake of heteroleptic bis (dipyrrinato) Zn complex **1d** at different concentration (1-10-20 µM in NIH3T3 cells (**panel 1**) and MCF7 cells (**panel 2**) without further staining. Intracellular accumulation was detected by using fluorescence confocal microscopy (Zeiss LSM800, Objective: C-Apo 40x 1.2 Water Immersion) λ_{exc} = 488 nm, λ_{em} 550-700nm. Scalebar: 25µm.

3.7.2 Cellular Uptake of complex 1e at different concentrations in NIH3T3 and MCF7 cells.

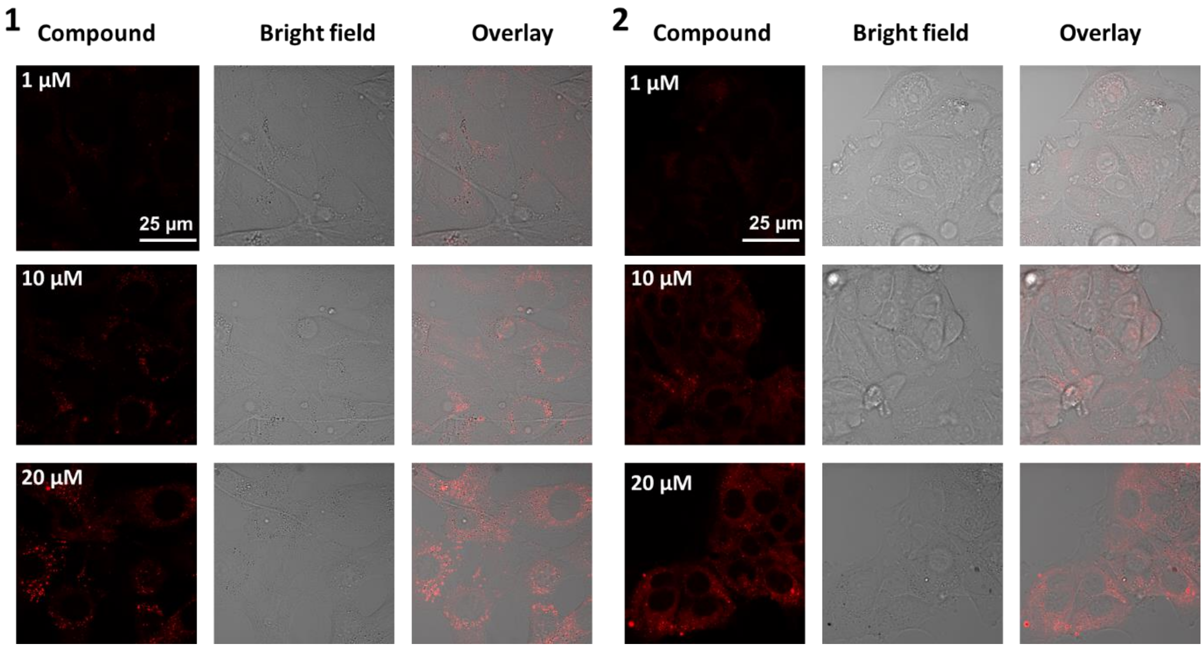

Figure S10: Cellular uptake of heteroleptic bis (dipyrrinato) Zn complex **1e** at different concentration (1-10-20 µM in NIH3T3 cells (**panel 1**) and MCF7 cells (**panel 2**) without further staining. Intracellular accumulation was detected by using fluorescence confocal microscopy (Zeiss LSM800, Objective: C-Apo 40x 1.2 Water Immersion) λ_{exc} = 488nm, λ_{em} 550-700nm. Scalebar: 25µm.

3.7.3 Cellular Uptake of complex 1a-e at 20 µM in NIH3T3 and MCF7 cells.

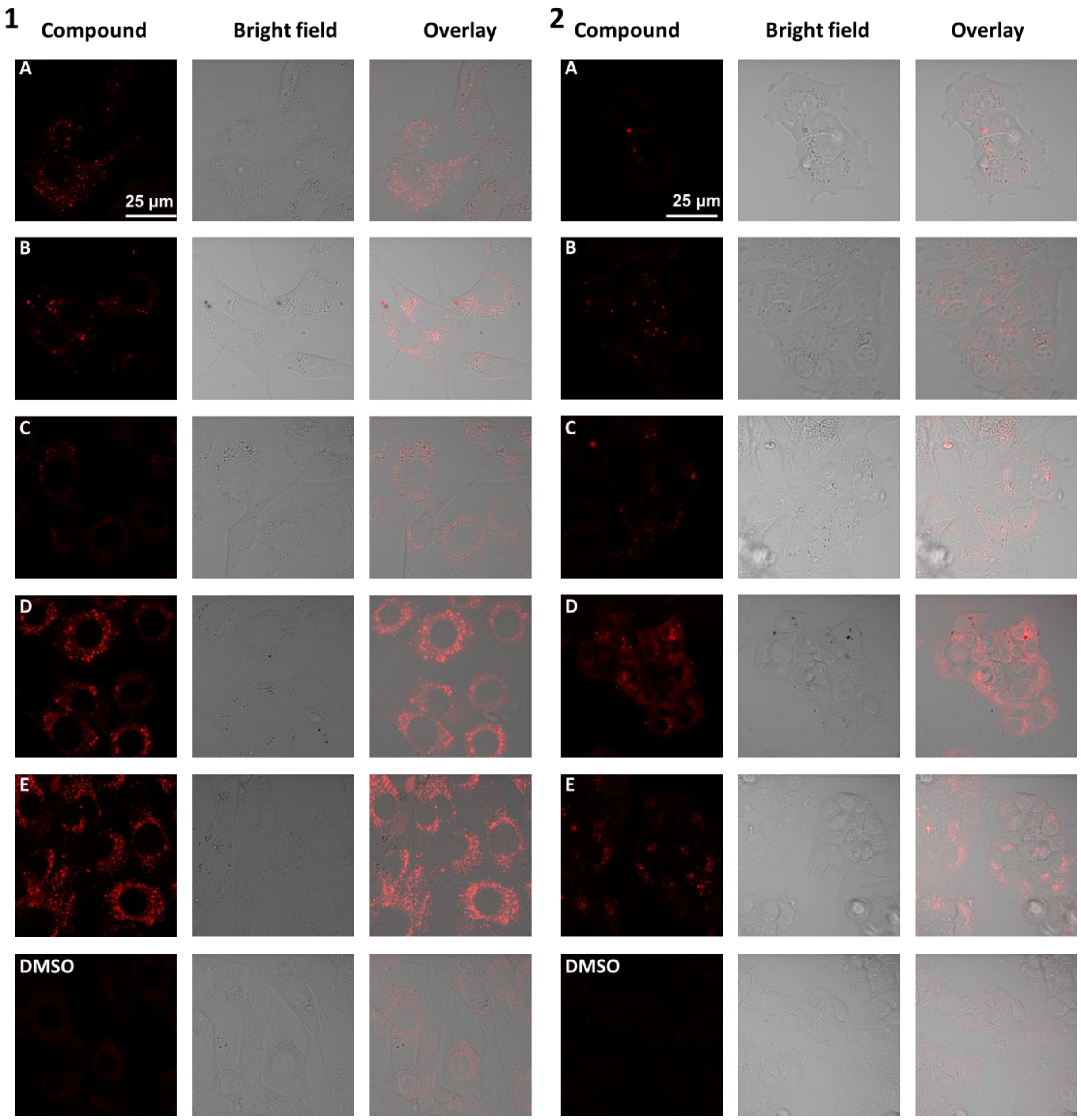

Figure **S11** : Cellular uptake of heteroleptic bis (dipyrrinato) Zn complexes **1a-e** (20µM) (A:**1a**, B:**1b**, C:**1c**, D:**1d**, E:**1e**) in NIH3T3 cells (**panel 1**) and MCF7 cells (**panel 2**) without further staining. Intracellular accumulation was detected by using fluorescence confocal microscopy (Zeiss LSM800, Objective: C-Apo 40x 1.2 Water Immersion) λ_{exc} = 488nm, λ_{em} 550-700nm. Scalebar: 25µm.

3.7.4 Intracellular co-localization experiments of complex 1a-e in HeLa cells.

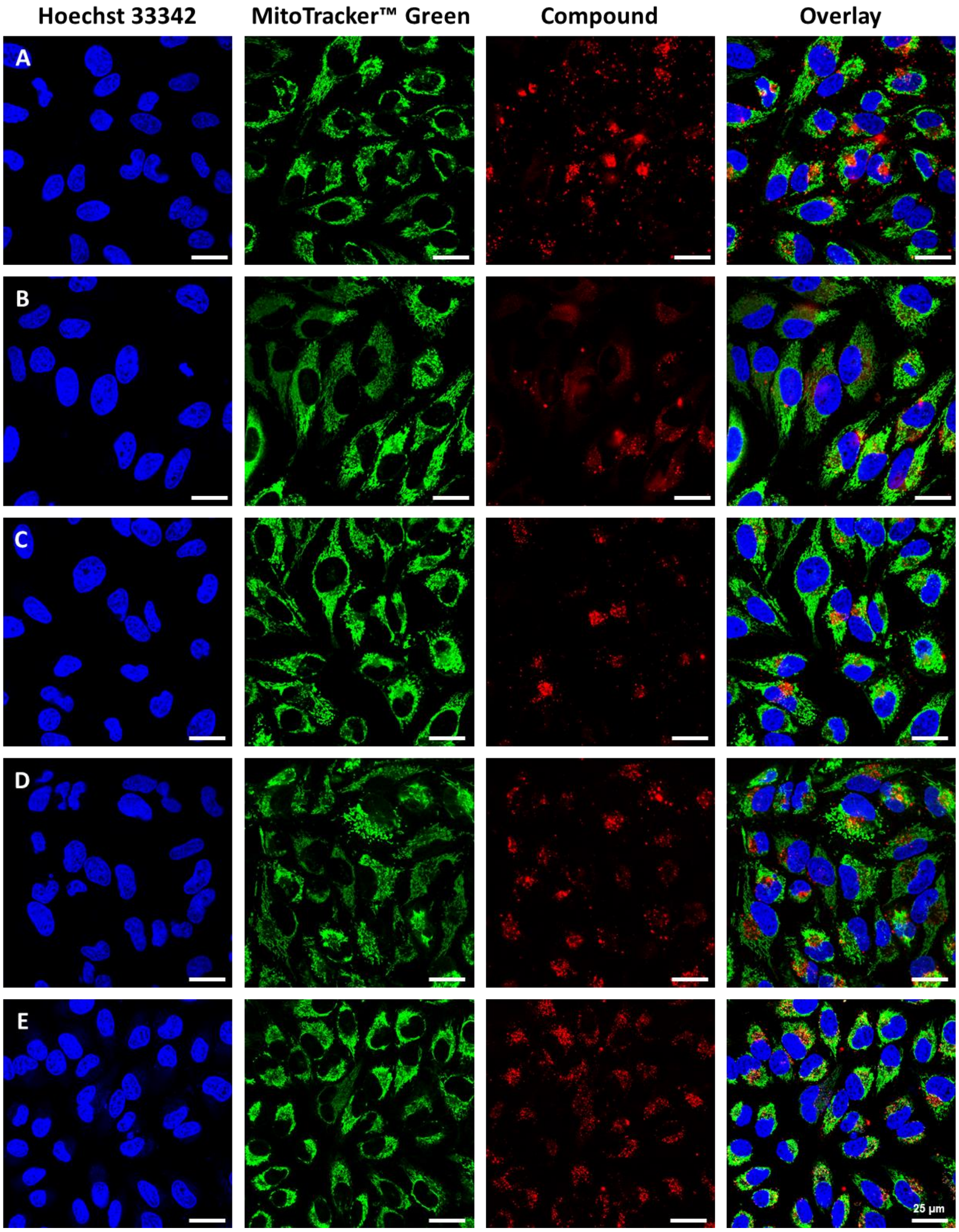

Figure **S12**: Cellular uptake of heteroleptic bis(dipyrrinato) zinc complexes **1a-e** (A:**1a**, B:**1b**, C:**1c**, D:**1d**, E:**1e**) in HeLa cells with MitoTracker™ Green. Intracellular accumulation was detected by using fluorescence confocal microscopy (Leica Stellaris, Objective: HC PL APO CS2 63x/1.40 OIL). From left to right: Hoechst 33342 (λ_{exc}=405nm, λ_{em}= 414-462 nm); MitoTracker™ Green (λ_{exc}=488 nm, λ_{em} 494-545 nm); compounds **1a-e** (λ_{exc} =630 nm, λ_{em} =640-750 nm). Scale bars 25 µm.

3.7.5 Intracellular co-localization experiments of complex 1a-e in NHDF cells.

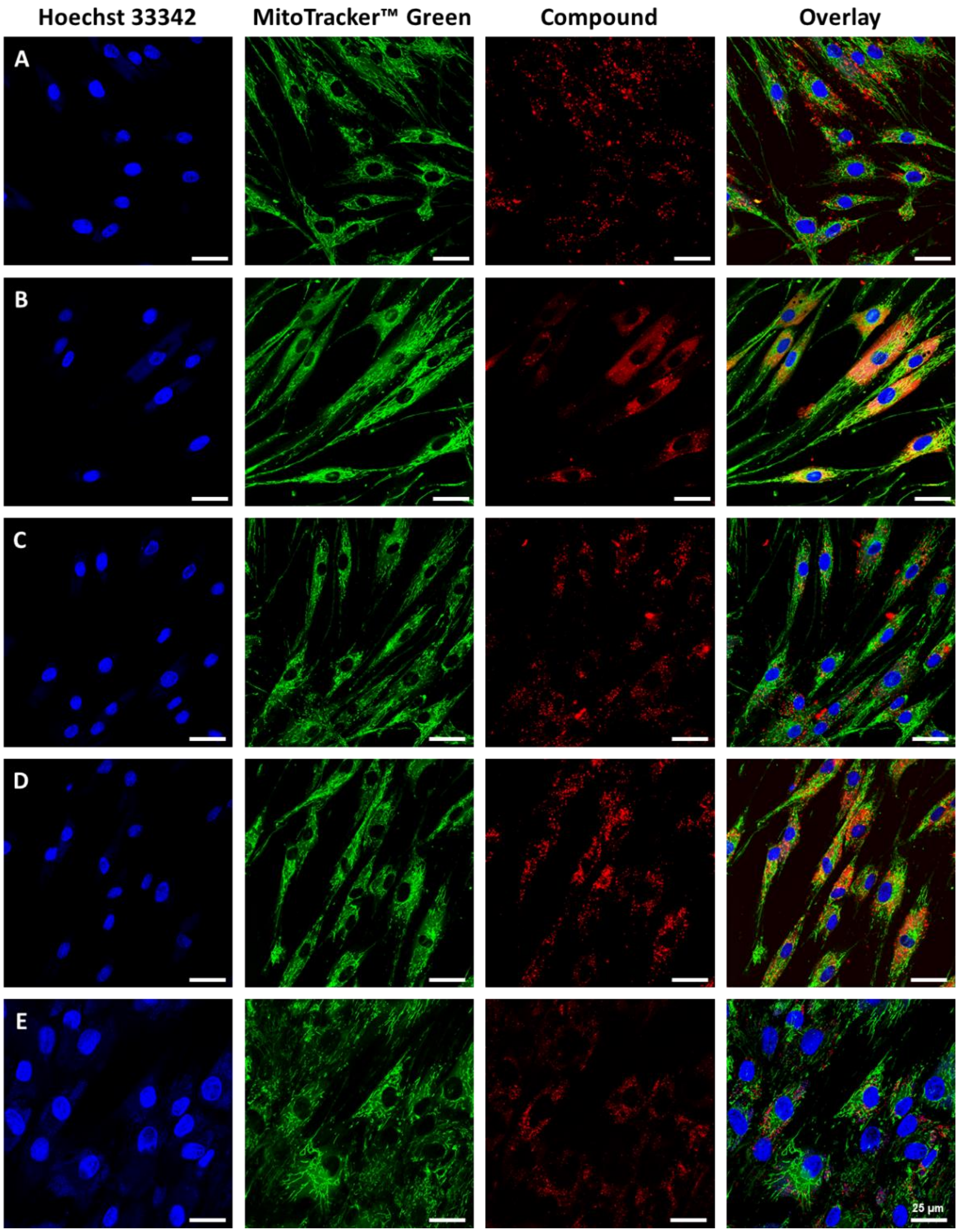

Figure **S13**: Cellular uptake of heteroleptic bis(dipyrrinato) zinc complexes **1a-e** (A:**1a**, B:**1b**, C:**1c**, D:**1d**, E:**1e**) in NHDF cells with MitoTracker™ Green. Intracellular accumulation was detected by using fluorescence confocal microscopy (Leica Stellaris, Objective: HC PL APO CS2 63x/1.40 OIL). From left to right: Hoechst 33342 (λ_{exc}=405nm, λ_{em}= 414-4462 nm); MitoTracker™ Green (λ_{exc}=488 nm, λ_{em} 494-545 nm); compounds **1a-e** (λ_{exc} =630 nm, λ_{em} =640-750 nm). Scale bars 25 µm.

3.7.6 Intracellular co-localization experiments of complex 1a-e in NIH3T3 cells.

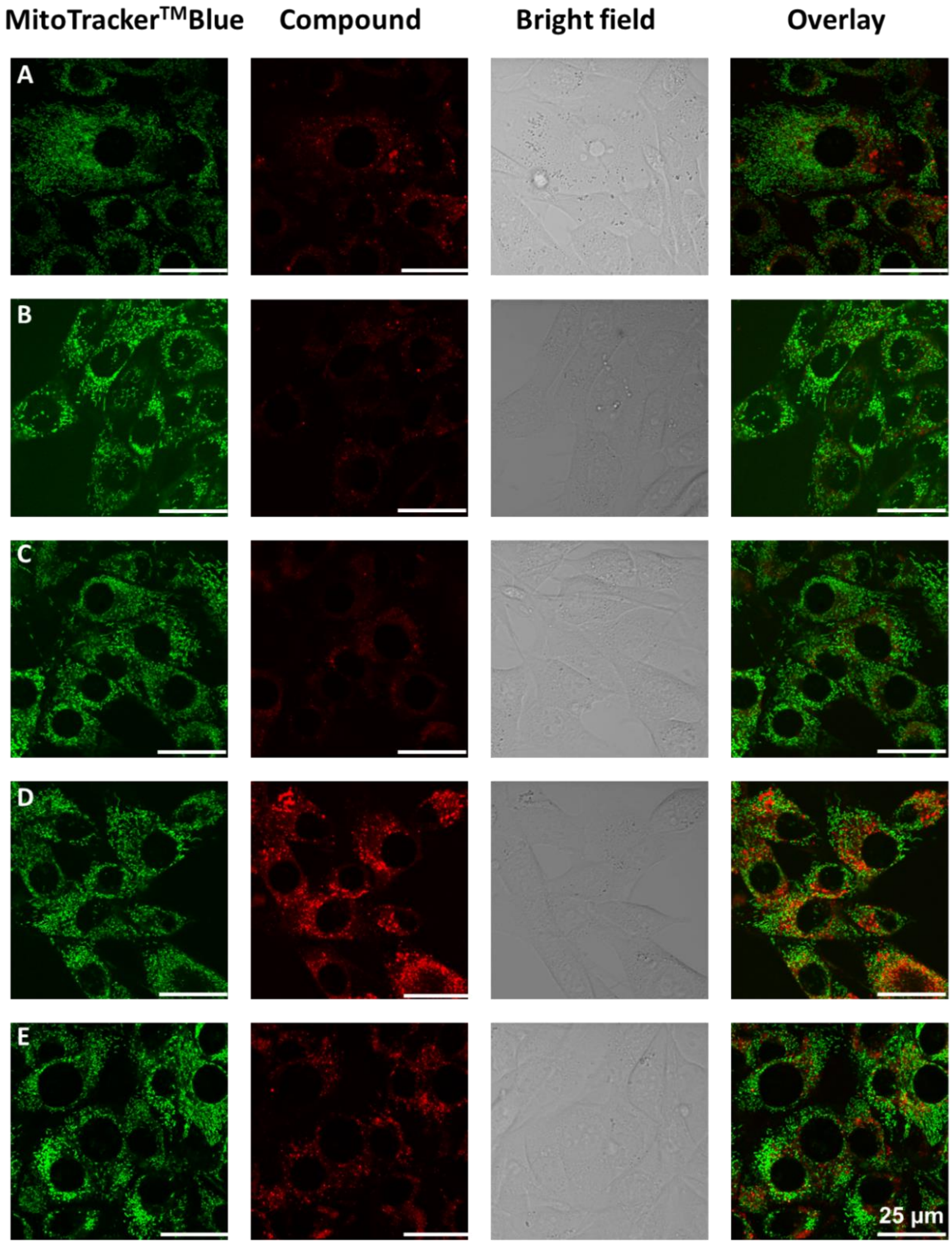

Figure **S14**: Cellular uptake of heteroleptic bis(dipyrrinato) zinc complexes **1a-e** (A:**1a**, B:**1b**, C:**1c**, D:**1d**, E:**1e**) in NIH3T3 cells with MitoTracker™ Blue. Intracellular accumulation was detected by using fluorescence confocal microscopy (Zeiss LSM800, Objective: C-Apo 40x 1.2 Water Immersion). From left to right: MitoTracker™ Blue (λ_{exc}=405 nm, λ_{em} 410-500 nm); compounds **1a-e** (λ_{exc} =488 nm, λ_{em} =550-700 nm). Scale bars 25 µm.

3.7.7 Intracellular co-localization experiments of complex 1a-e in HeLa cells.

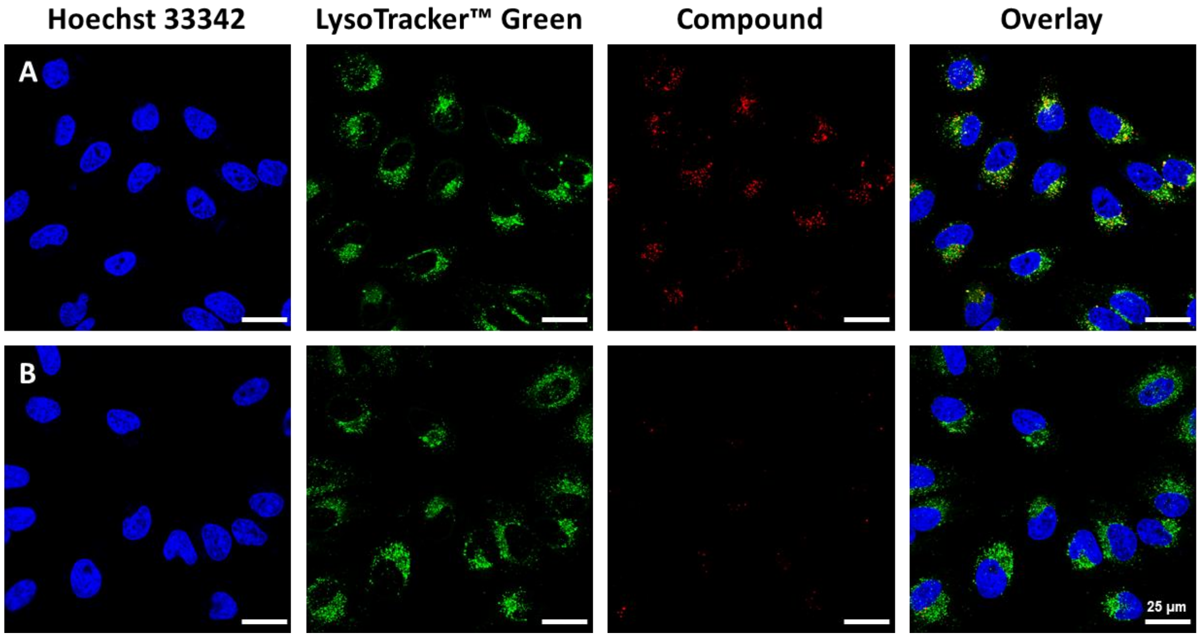

Figure **S15**: Cellular uptake of heteroleptic bis(dipyrrinato) zinc complexes **1b** and **1c** (A:**1b**, B:**1c)**. (Complexes **1a**, **1d**, **1e** are reported in Fig. 5 of the manuscript) in HeLa cells with LysoTracker™ Green. Intracellular accumulation was detected by using fluorescence confocal microscopy (Leica Stellaris, Objective: HC PL APO CS2 63x/1.40 OIL). From left to right: Hoechst 33342 (λ_{exc}=405nm, λ_{em}= 414-462 nm); LysoTracker™ Green (λ_{exc}=488 nm, λ_{em} 494-545 nm); compounds **1a-e** (λ_{exc} =630 nm, λ_{em} =640-750 nm). Scale bars 25 µm.

3.7.8 Intracellular co-localization experiments of complex 1a-e in NIH3T3 cells.

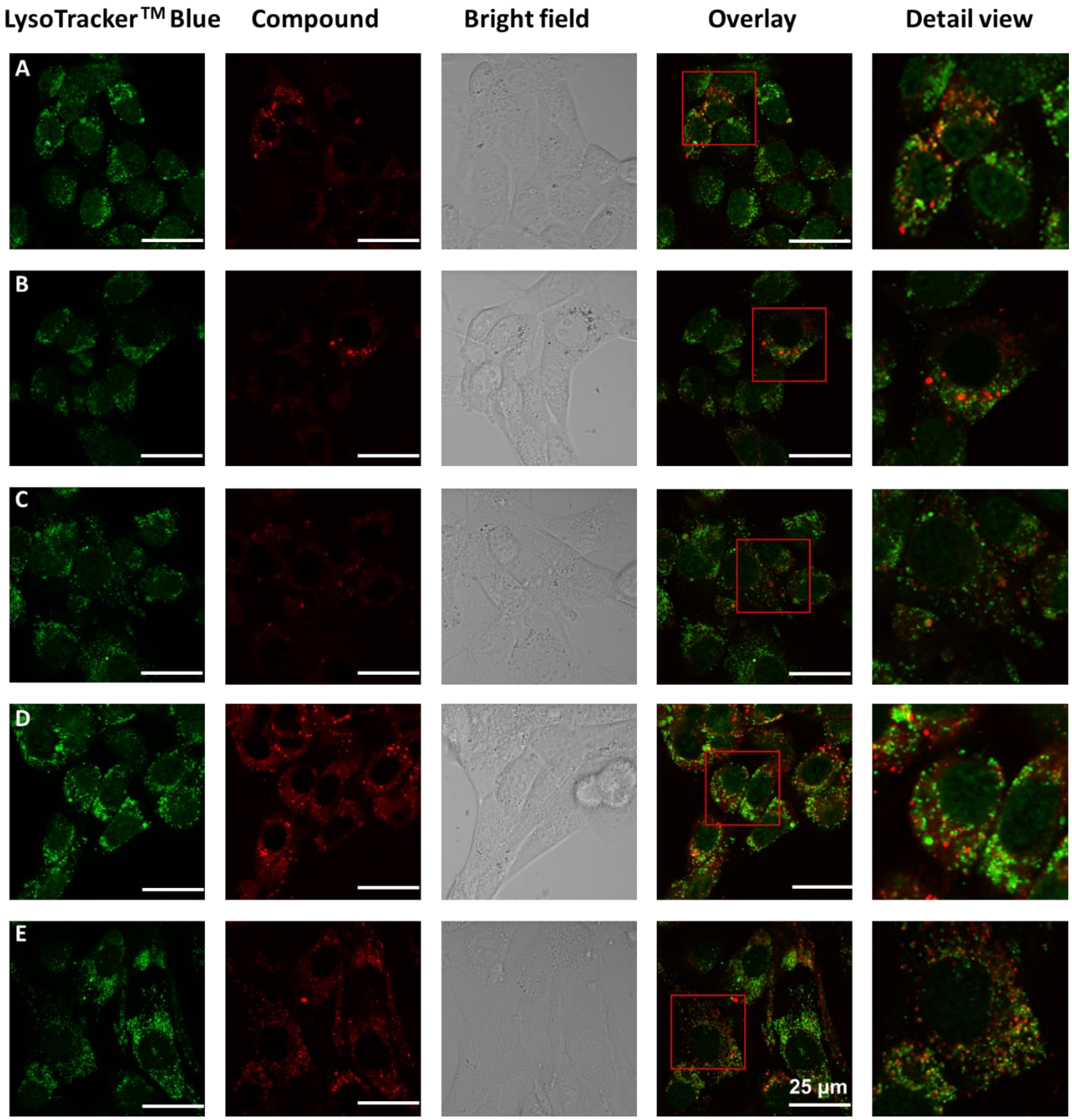

Figure **S16**: Cellular uptake of heteroleptic bis(dipyrrinato) zinc complexes **1a-e** (A:**1a**, B:**1b**, C:**1c**, D:**1d**, E:**1e**) in NIH3T3 cells with LysoTracker™ Blue. Intracellular accumulation was detected by using fluorescence confocal microscopy imaging parameters for the compounds (Zeiss LSM800, Objective: C-Apo 40x 1.2 Water Immersion). From left to right: LysoTracker™ Blue (λ_{exc}=402 nm, λ_{em} 410-500 nm); compounds **1a-e** (λ_{exc} =488 nm, λ_{em} =550-700 nm). Scale bars 25 µm.

3.7.9 Cellular uptake over time of complexes 1a-e in NIH3T3 and MCF7 cells.

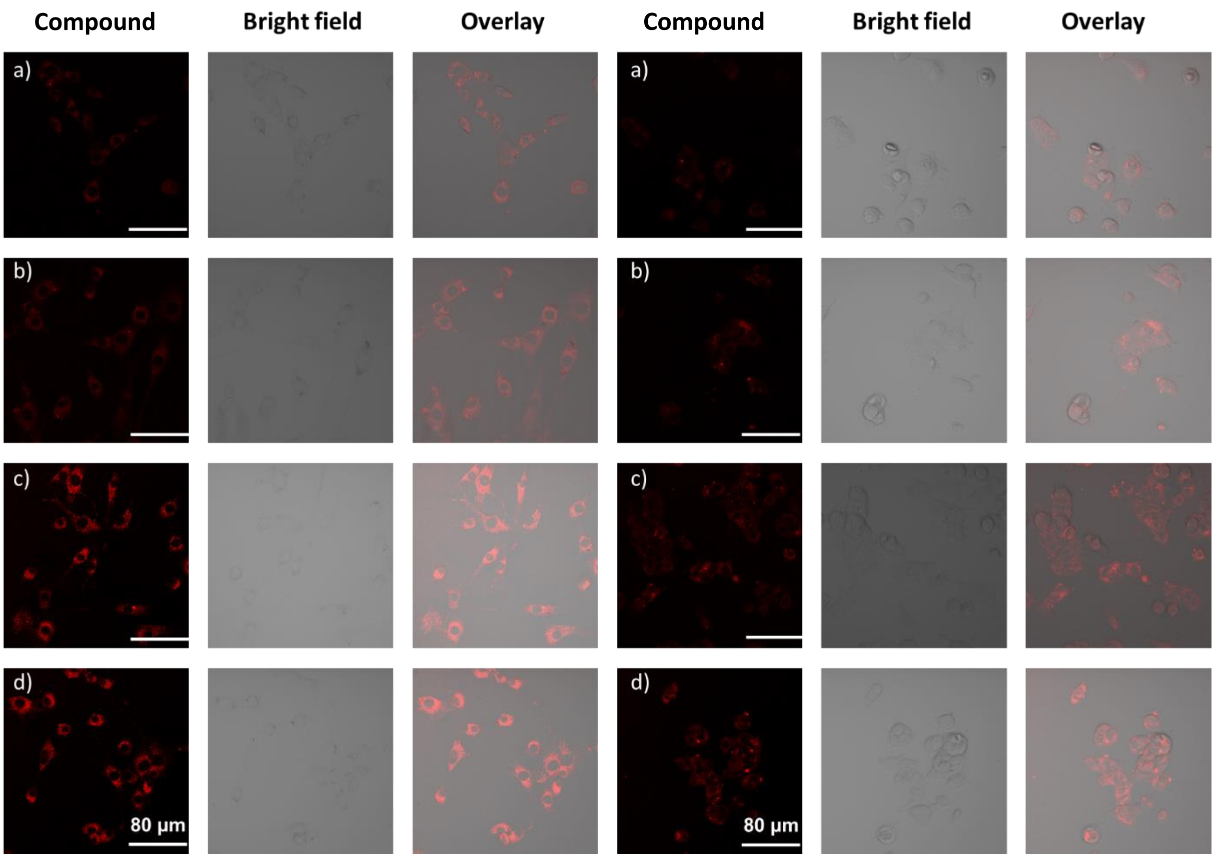

Figur**e S17**: Cellular uptake of heteroleptic bis (dipyrrinato) Zn complex **1e** (20µM) in NIH3T3 cells (**panel 1**) and MCF7 cells (**panel 2**) over time (a: 0.5 h; b: 1.5 h; c:6h) and post fixation (d). After 6 h, samples were fixed and incubated for further 24 h and then imaged (d). Intracellular accumulation was detected by using fluorescence confocal microscopy (Zeiss LSM800, Objective: PLN-Apo 20x 0.8 AIR) (λ_{exc} = 488nm, λ_{em} 550-700nm). Scalebar: 80 µm.

4 ^{1}H-NMR and ^{13}C-NMR spectra of novel complexes.

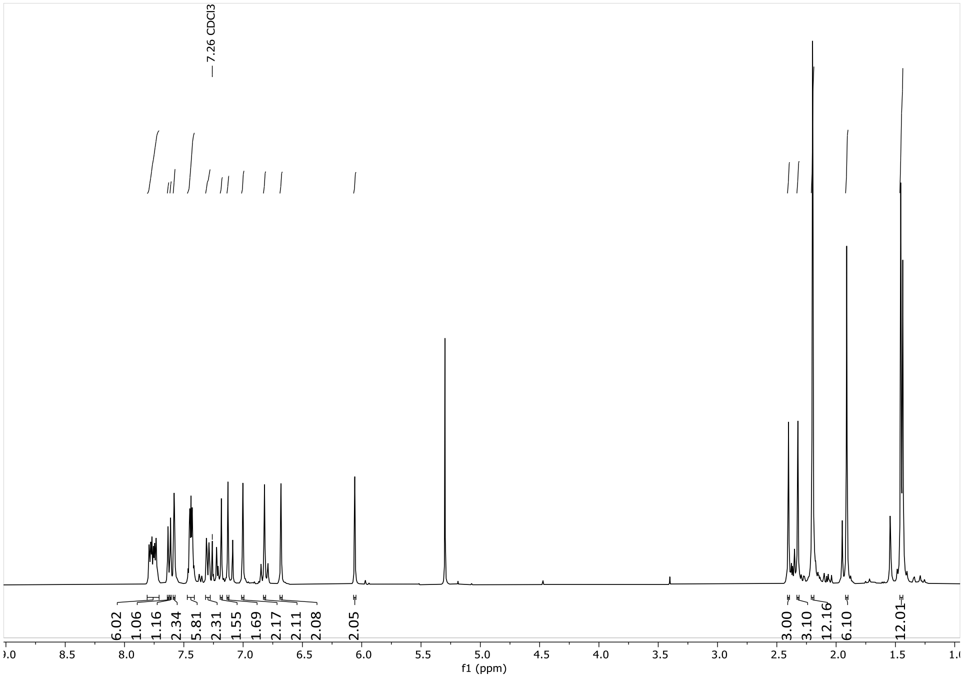

Figure **S18**: ^{1}H-NMR of **1a** in $CDCl_3$ (residual solvent peaks: 1.56 ppm (s, water), 2.07 (ethyl acetate).

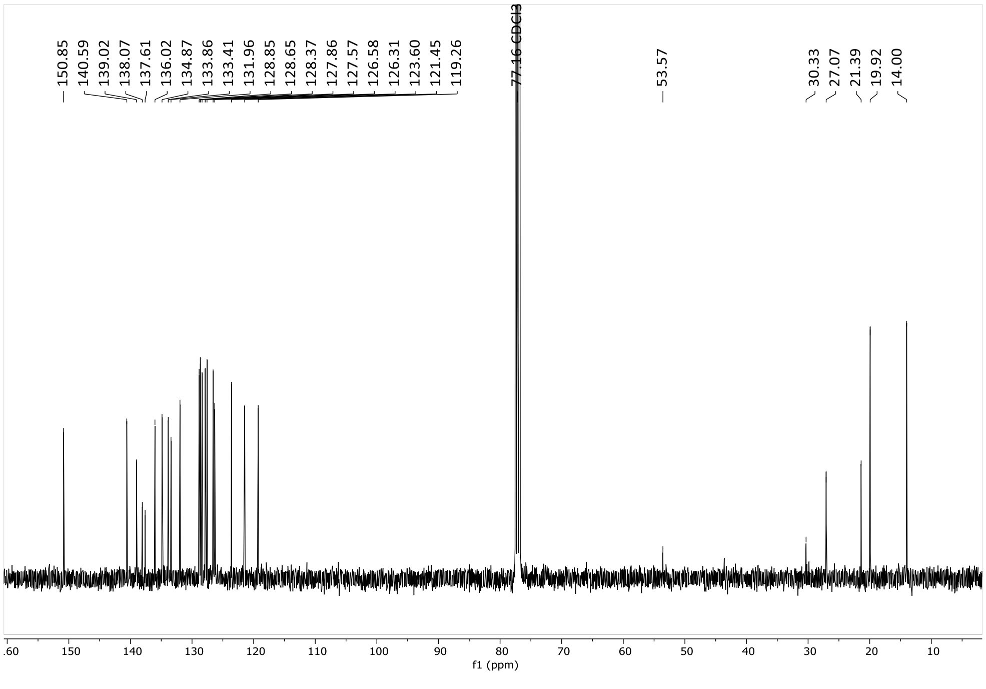

Figure **S19**: ^{13}C-NMR of **1a** in $CDCl_3$ (residual solvent peak: 53.57 (dichloromethane)).

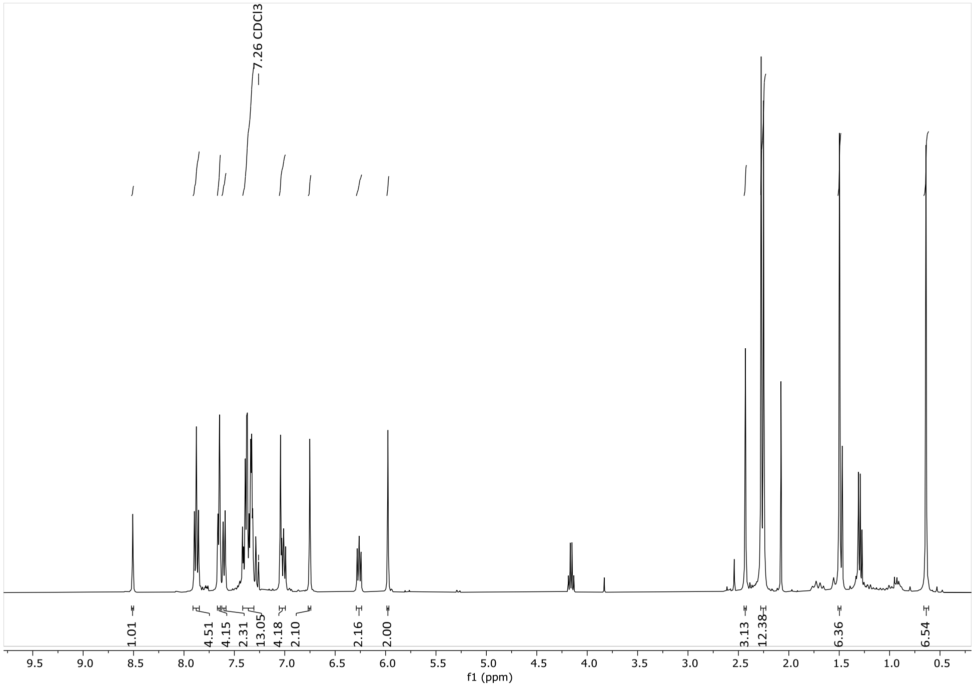

Figure **S20**: ^{1}H-NMR of **1b** in $CDCl_3$ (residual solvent peaks: 1.47 ppm (cyclohexane), 4.08 ppm (q, ethyl acetate).

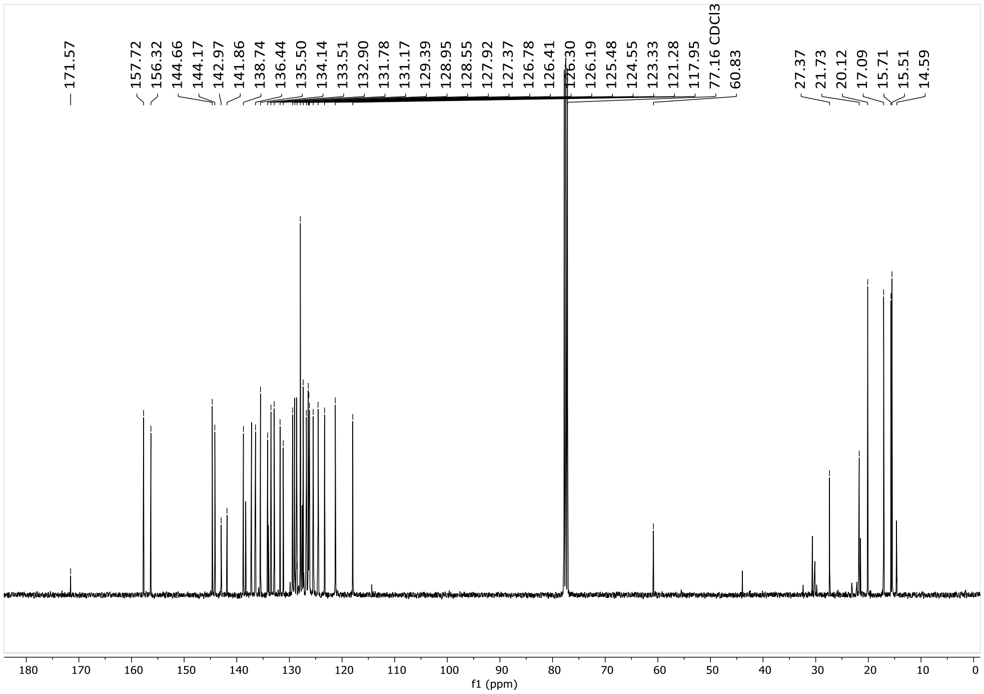

Figure **S21**: ^{13}C-NMR of **1b** in $CDCl_3$ (residual solvent peaks: 171.57 ppm, 60.82 ppm, 14.59 ppm (ethyl acetate).

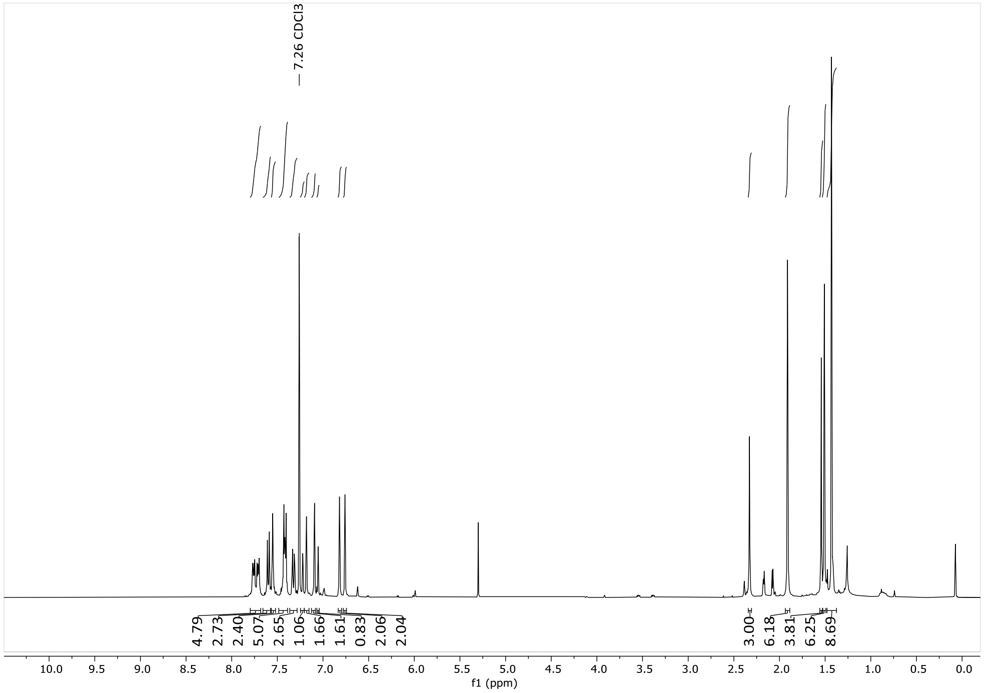

Figure **S22**: ^{1}H-NMR of **1c** in $CDCl_3$ (residual solvent peaks: 1.2 (ethyl acetate), 5.29 (s, dichloromethane).

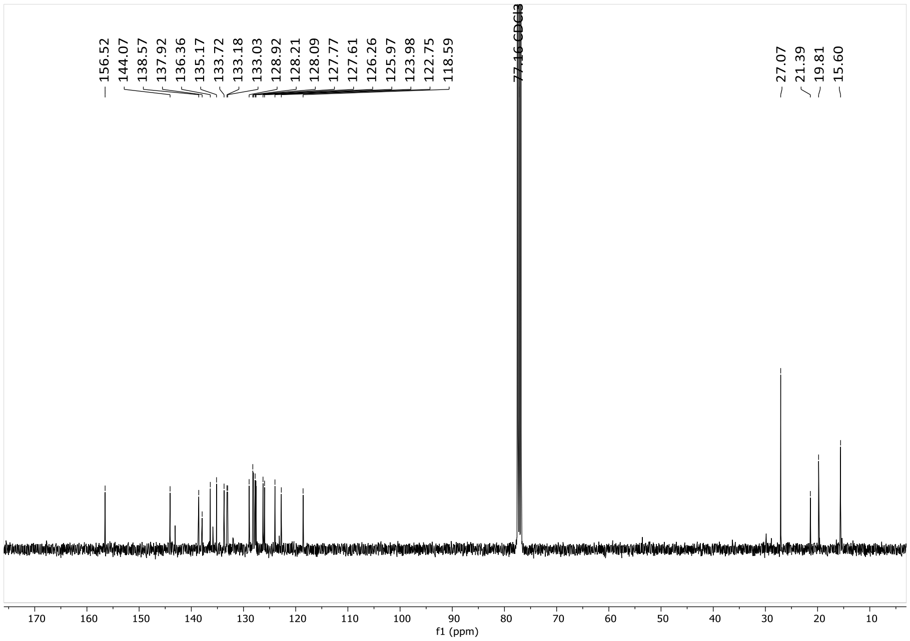

Figure **S23**: ^{13}C-NMR of **1c** in $CDCl_3$.

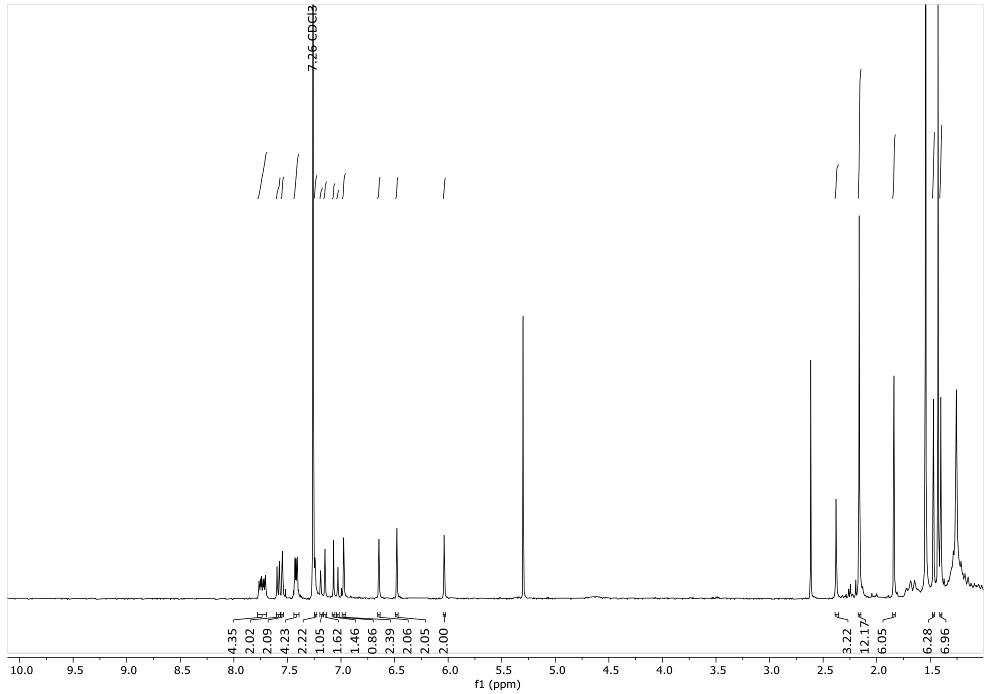

Figure **S24**: ^{1}H-NMR of **1d** in $CDCl_3$ (residual solvent peaks: 1.2 (ethyl acetate), 2.6 (s, dimethyl sulfoxide), 5.29 (s, dichloromethane).

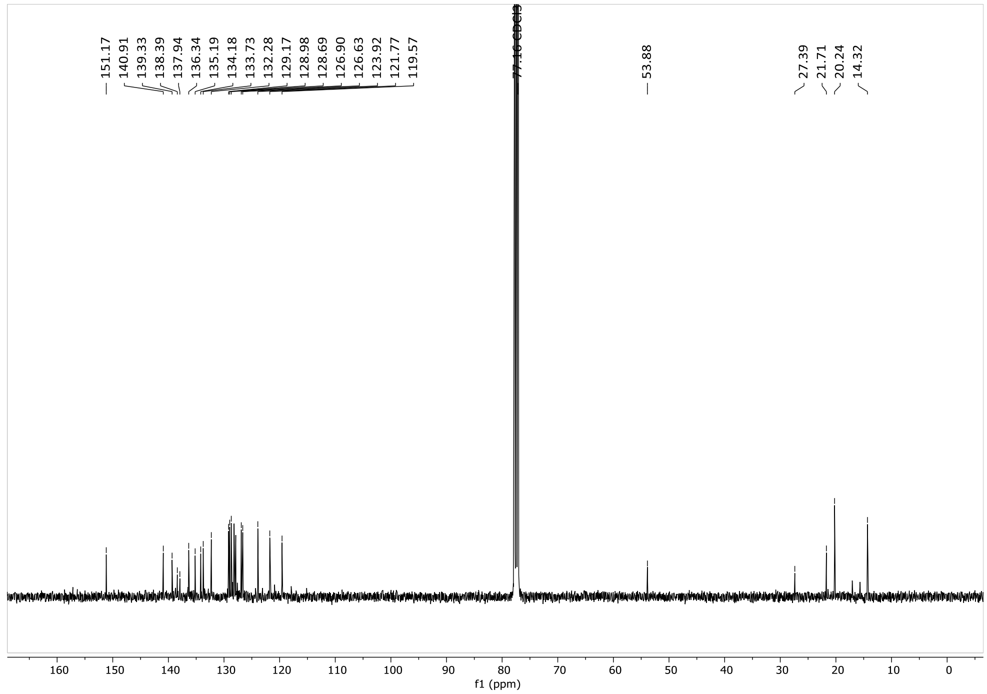

Figure **S25**: ^{13}C-NMR of **1d** in $CDCl_3$.

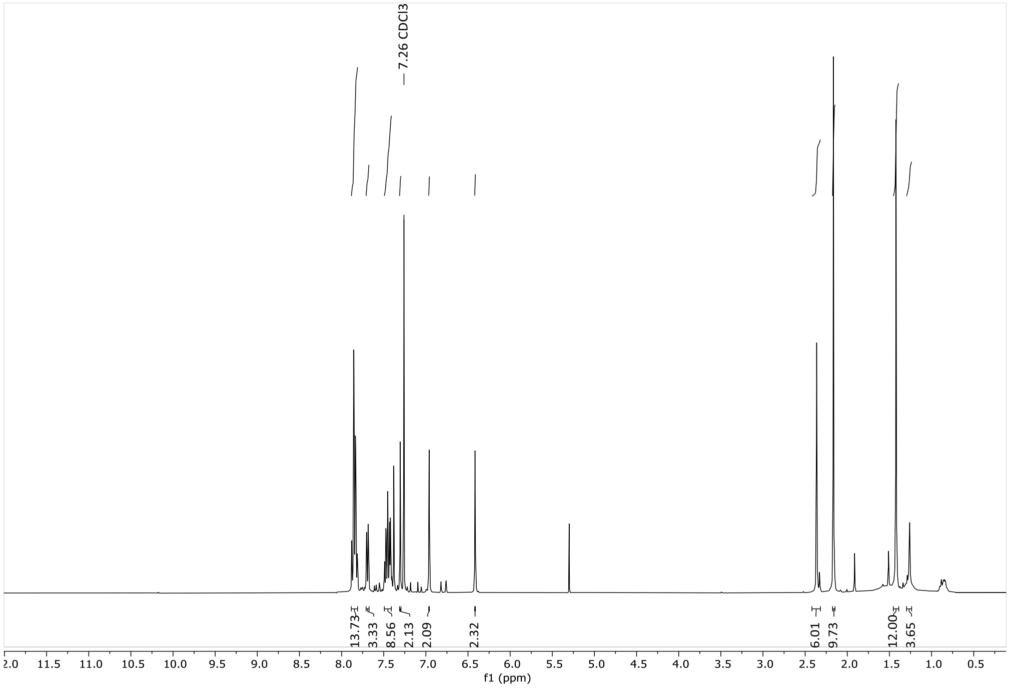

Figure **S26**: ^{1}H-NMR of **1e** in $CDCl_3$ (residual solvent peaks: 1.5 (water), 2.6 (s, dimethyl sulfoxide), 5.29 (s, dichloromethane).

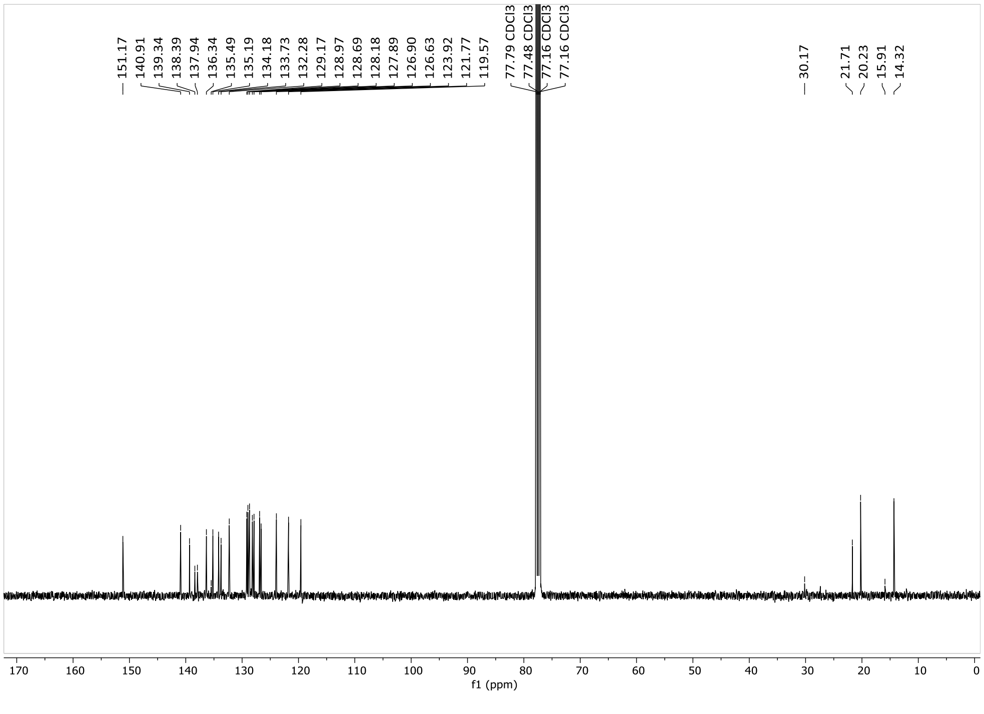

Figure **S27**: ^{13}C-NMR of **1e** in $CDCl_3$

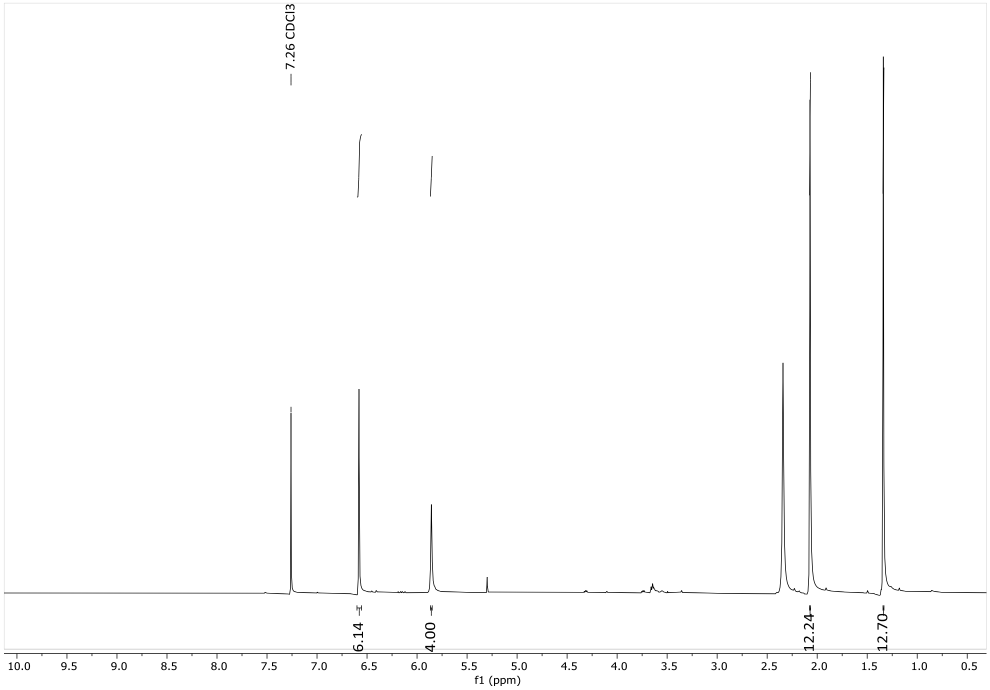

Figure 28:^{1}H-NMR of **2c** in $CDCl_3$ (residual solvent peaks: 2.6 (s, dimethyl sulfoxide).

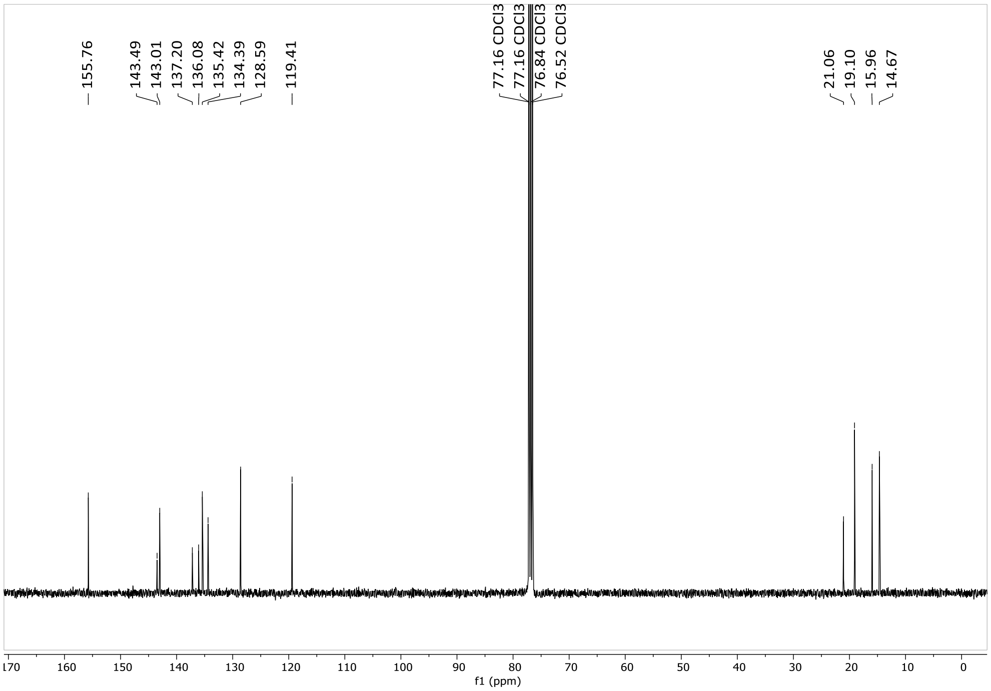

Figure 29: ^{13}C-NMR of **2c** in $CDCl_3$.

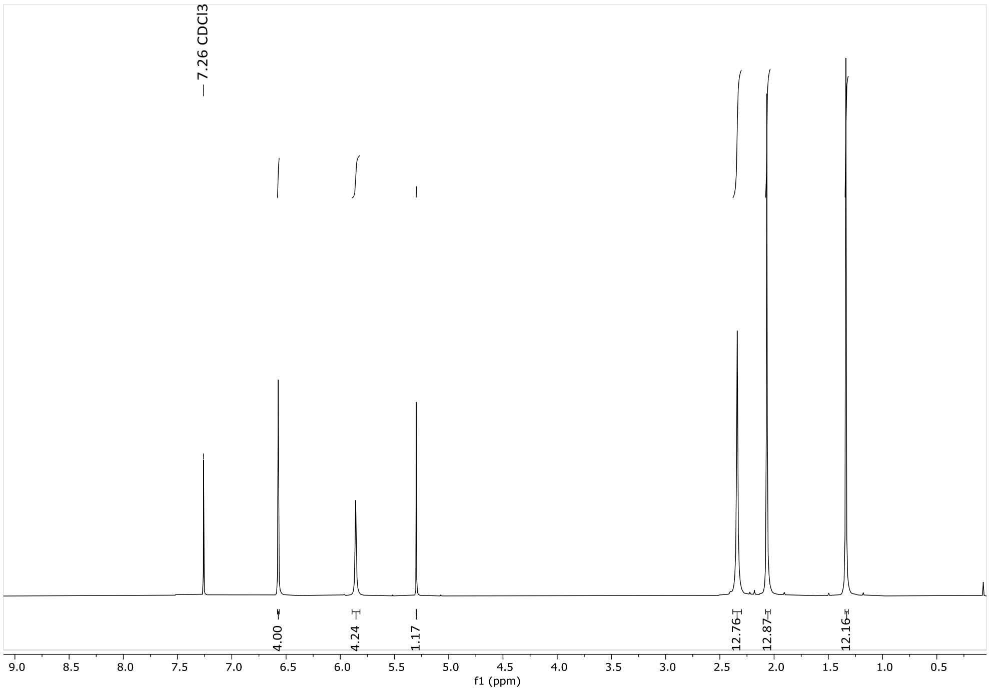

Figure **S30**:^{1}H-NMR of **2d** in $CDCl_3$.

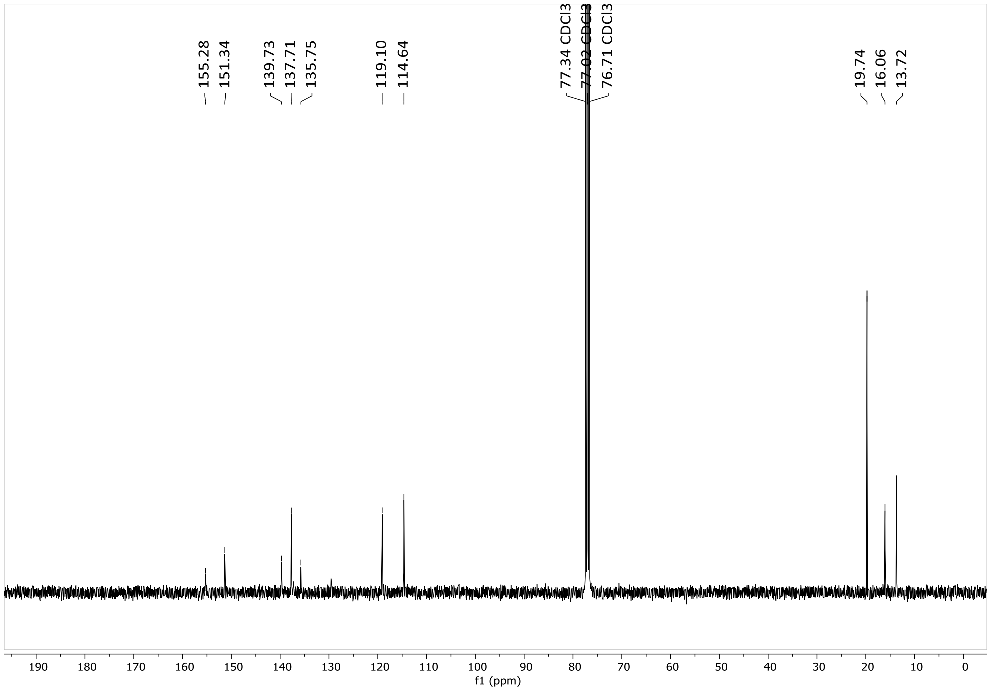

Figure **S31**: ^{13}C-NMR of **2d** in $CDCl_3$.

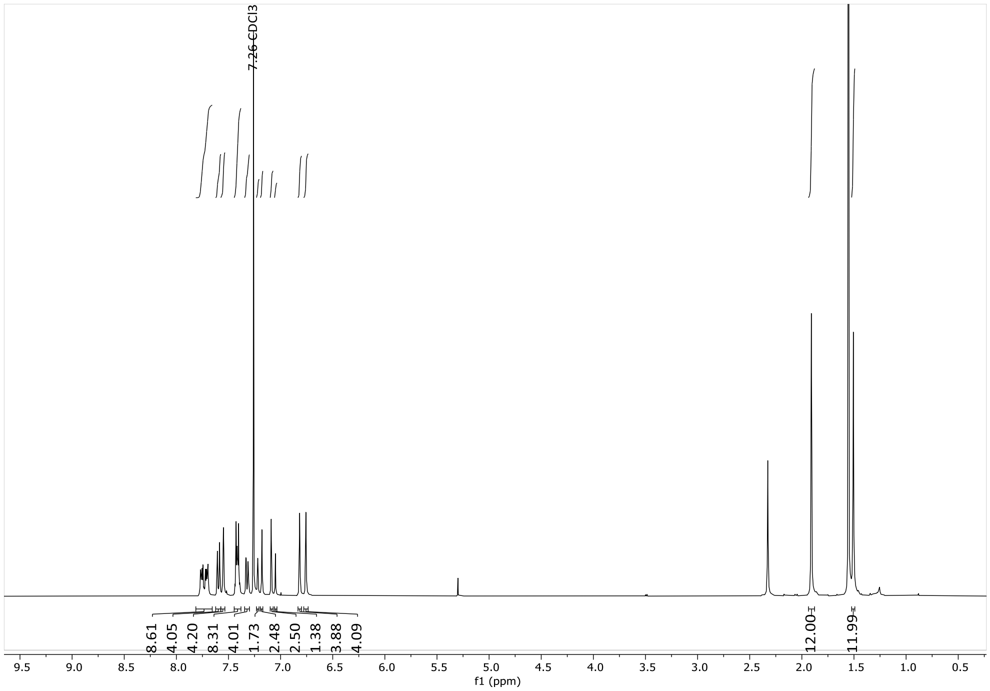

Figure S32: ^{1}H-NMR of **3e** in $CDCl_3$ (residual solvent peaks: 1.2 (ethyl acetate), 2.6 (s, dimethyl sulfoxide), 5.29 (s, dichloromethane).

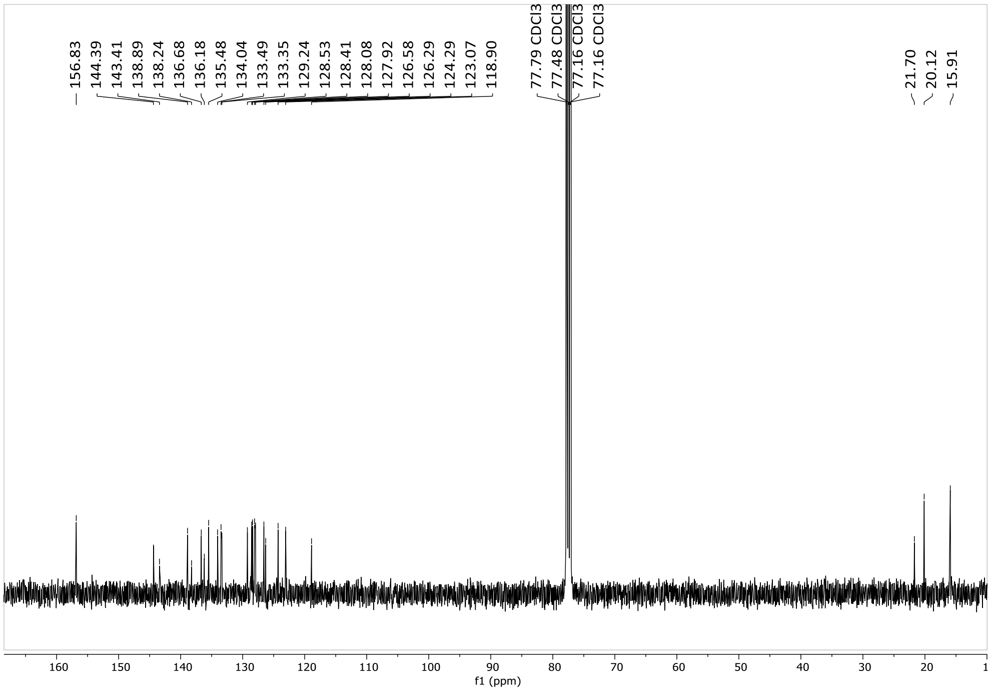

Figure **S33**: ^{13}C-NMR of **3e** in $CDCl_3$.

References

Bolte, S., and Cordelières, F.P. (2006). A guided tour into subcellular colocalization analysis in light microscopy. *Journal of Microscopy* 224**,** 213-232.

Brouwer, A.M. (2011). Standards for photoluminescence quantum yield measurements in solution (IUPAC Technical Report). *Pure and Applied Chemistry* 83**,** 2213-2228.

Dalessandro, E.V., Collin, H.P., Guimaraes, L.G.L., Valle, M.S., and Pliego, J.R., Jr. (2017). Mechanism of the Piperidine-Catalyzed Knoevenagel Condensation Reaction in Methanol: The Role of Iminium and Enolate Ions. *J Phys Chem B* 121**,** 5300-5307.

I.V. Sazanovich, C.K., E. Hindin, L. Yu, D. F. Bocian, J. S. Lindsey, D.Holten <J.Am.Chem.Soc. 2004, 126, Structural Control of the excited state of bis dipyrrinato zinc complexes.pdf>.

Mani, V., Krishnakumar, V.G., Gupta, S., Mori, S., and Gupta, I. (2017). Synthesis and characterization of styryl-BODIPY derivatives for monitoring in vitro Tau aggregation. *Sensors and Actuators B: Chemical* 244**,** 673-683.

Sakamoto, R., Iwashima, T., Kogel, J.F., Kusaka, S., Tsuchiya, M., Kitagawa, Y., and Nishihara, H. (2016). Dissymmetric Bis(dipyrrinato)zinc(II) Complexes: Rich Variety and Bright Red to Near-Infrared Luminescence with a Large Pseudo-Stokes Shift. *J Am Chem Soc* 138**,** 5666-5677.

Tsuchiya, M., Sakamoto, R., Shimada, M., Yamanoi, Y., Hattori, Y., Sugimoto, K., Nishibori, E., and Nishihara, H. (2016). Bis(dipyrrinato)zinc(II) Complexes: Emission in the Solid State. *Inorg Chem* 55**,** 5732-5734.

Tungulin, D., Leier, J., Carter, A.B., Powell, A.K., Albuquerque, R.Q., Unterreiner, A.N., and Bizzarri, C. (2019). Chasing BODIPY: Enhancement of Luminescence in Homoleptic Bis(dipyrrinato) Zn(II) Complexes Utilizing Symmetric and Unsymmetrical Dipyrrins. *Chemistry* 25**,** 3816-3827.
